# The anti-biofilm compound 4-ethoxybenzoic acid inhibits *Staphylococcus aureus* virulence factor production via a putative 4EB-binding pocket in key virulence-associated proteins

**DOI:** 10.3389/fmicb.2025.1704290

**Published:** 2026-02-02

**Authors:** Caroline C. Taylor, Adonis Aviles-Gonzalez, Alexander Marchesani, Christina Kiessling, Travis Patrick, Linxin Chen, Haozhe Yao, Zixuan Li, Abbie Seward, Kuk-Jeong Chin, Eric S. Gilbert

**Affiliations:** Department of Biology, Georgia State University, Atlanta, GA, United States

**Keywords:** 4-ethoxybenzoic acid, antimicrobial resistance, anti-virulence, biofilm, infectious disease, *Staphylococcus aureus*

## Abstract

There is a need for dual action anti-virulence and anti-biofilm agents that target the opportunistic pathogen *Staphylococcus aureus*. Previous research determined that 0.8 mg/mL 4-ethoxybenzoic acid (4EB) reduced *S. aureus* ATCC 6538 biofilm formation by 88% relative to untreated controls with moderate inhibition of planktonic cell growth. Here we report that 4EB impacted *S. aureus* virulence phenotypes across all growth phases, including alpha-hemolysin (Hla) and serine protease (SplB/C) exoprotein production (60% reduction), staphyloxanthin pigment accumulation (73% reduction) and alpha-hemolysis (>87% reduction) compared to untreated control cells. RT-qPCR analysis demonstrated that 4EB downregulated virulence gene expression, including >100-fold reduction of alpha-hemolysin (*hla*) and leukocidins (*lukDvEv*), and a 35-fold decrease of the response regulator SaeR. Phenol-soluble modulin (PSM) transcription by biofilm-grown cells was upregulated by 4EB more than 4-fold for α1-4*psm* and β1-2*psm* genes, while *δ*-toxin (*hld*) was unaffected. *In silico* molecular docking analysis revealed that 4EB has a strong binding affinity (ΔG < −6.0 kcal/mol) for 9 virulence-associated transcriptional regulators, including SaeS, IcaR and CodY. Analysis of gene transcription during late exponential phase growth determined that genes controlled by 7 of the 9 identified regulators were significantly impacted by 4EB. The docking analysis identified putative 4EB binding sites that share common features including valine and tyrosine amino acid residues. The combined *in vitro* and *in silico* analyses identified interactions with well-known virulence genes but also implicated an effect of 4EB on proteins less commonly associated with *S. aureus* pathogenesis. These findings suggested potential alternative targets for anti-virulence and anti-biofilm therapeutics.

## 1 Introduction

*Staphylococcus aureus* exists as both a commensal bacterium and a human pathogen and is a leading cause of hospital- and community-acquired pneumonia, skin and soft-tissue infections, and biofilm-associated endocarditis (Tong et al., 2015; Kourtis et al., 2019). The non-systemic *S. aureus* infections typically involve biofilm production, which helps the bacterium evade the host immune system and provides a physical barrier to resist antibiotics (Weiner et al., 2016). Additionally, *S. aureus* contains a plethora of virulence factors that aid in disease progression. These include, but are not limited to, toxins, exoproteins, and pigments (Paharik et al., 2016; Burlak et al., 2007; Schilcher et al., 2024; Vila et al., 2019). Secreted virulence factors are typically produced post-exponentially, advancing the infection process by lysing host immune cells, dysregulating the immune system, and activating T cells (Kim et al., 2021; Chen et al., 2022). The virulence of *S. aureus* has earned it a spot among the ESKAPE pathogens, all of which are global health threats (De Oliveira et al., 2020).

*Staphylococcus aureus* infections have become increasingly problematic because of the emergence of multi-drug resistance. Resistance to beta-lactam antibiotics is virtually omnipresent in *S. aureus* clinical isolates (Lowy, 2003), and some isolates are resistant to almost all antibiotics that are clinically available, including vancomycin, considered the “last resort” antibiotic (Cheung et al., 2021). The prevalence of resistant strains has accelerated a push to find alternative treatments that are non-bactericidal and generate less selective pressure than antibiotic therapies (Cheung et al., 2021). Several anti-virulence compounds to control *S. aureus* infections were selected to target the *agr* quorum sensing system, which regulates production of virulence phenotypes, including biofilm formation and hemolysis (Sully et al., 2014; Mahdally et al., 2021). A challenge with this strategy exists because inhibiting *agr* quorum sensing in *S. aureus* leads to increased biofilm formation, a behavior observed in *agr*-deficient clinical isolates obtained from antibiotic-resistant biofilm infections (He et al., 2023; Otto, 2023). Thus, a need exists for additional *S. aureus* therapeutics with both anti-virulence and anti-biofilm activity.

In previous research, we identified small molecules from *Rhamnus prinoides*, an East African shrub used in traditional medicine, that exhibited anti-virulence and anti-biofilm activity (Campbell et al., 2019). Further investigation identified 4-ethoxybenzoic acid (4EB), which reduced biofilm formation up to 88% relative to untreated controls with moderate impact on planktonic growth (Campbell et al., 2020). These findings align with other reports in the literature of small molecule natural products that elicit both anti-biofilm and antimicrobial activity (Campbell et al., 2024; Khodaverdian et al., 2013; Qiu et al., 2010). We hypothesized that their small size allows these molecules to interact with multiple virulence-associated proteins, including transcriptional regulators. Accordingly, we hypothesized that 4EB modifies additional virulence phenotypes beyond biofilm formation. In this work, we investigated the impact of 4EB on *S. aureus* virulence factor production and used molecular docking to assess the ability of 4EB to interact with *S. aureus* transcriptional regulators and select virulence-associated proteins.

## 2 Methods

### 2.1 *In vitro* assays

#### 2.1.1 Chemicals

All chemicals used in this work, including 4EB, were obtained from Millipore Sigma (United States) unless otherwise indicated.

#### 2.1.2 Bacterial strain and culturing conditions

*Staphylococcus aureus* ATCC 6538 was grown in Luria-Bertani (LB) broth (Becton Dickinson, United States) and was used in all experiments. Prior to each experiment, overnight cultures were prepared by inoculating cells (stored in 10% glycerol at −80 °C) into sterile LB broth and incubating with aeration for 14–16 h at 37 °C, 200 rpm. For experiments involving 4EB, the concentration of 4EB was 0.8 mg/mL unless otherwise indicated. 4EB was aseptically weighed and added directly into LB broth prior to inoculation. Inocula for experiments were prepared as follows: an overnight culture was grown, the optical density (OD_600_) was measured, and sample cultures were prepared by inoculating sterile LB broth to an OD_600_ of 0.01, with or without 4EB in the growth medium.

#### 2.1.3 Biofilm imaging

Biofilms were cultivated in flows cells suitable for imaging by confocal laser scanning microscopy (CLSM). Details on flow cell construction, biofilm cultivation and imaging can be found in the Supplementary materials. Briefly, biofilms were grown for 24 h in continuous flow conditions with or without 4EB, were stained with acridine orange and were imaged using an LSM510 CLSM (Zeiss) at 100 × magnification. Zeiss Zen Lite software was used to visualize and quantify the biofilms. The data presented are the average pixel intensity measured by the microscope for the collected images.

#### 2.1.4 Exoprotein extraction procedure

Exoproteins were isolated as described (Traber and Novick, 2006) with modifications. Cultures were grown for 15 h (early stationary growth phase) at 37 °C, 200 rpm. At 15 h, the cultures were placed on ice for 1 h, and then the OD_600_ of each 4EB-treated culture was normalized to the untreated culture so that each treated and untreated culture had approximately the same number of cells (OD_600_ = 6.0 to 6.5). Culture supernatants were collected by centrifugation at 4,000 x *g* for 20 min at 4 °C, followed by filtering through 0.22 μm membrane filters to remove cell debris. Exoproteins were precipitated overnight at 4 °C using 1:1 (v/v) ice-cold 10% trichloroacetic acid added into the filtrate. The following day, samples were centrifuged at 4,000 x *g* at 4 °C for 20 min, and the protein precipitate was washed with 95% ethanol and air-dried at 4 °C. The protein precipitate was subsequently resuspended in 1x sample buffer (NuPAGE™ LDS Sample Buffer) and stored at −20 °C until needed for exoprotein profiling.

#### 2.1.5 Exoprotein profiling

Exoproteins were profiled using the NuPAGE™ Mini Protein System (Invitrogen, United States). Exoprotein extracts were reduced and incubated at 70 °C for 10 min. The exoproteins were profiled on Bis-Tris Mini Gels (4–12%) using MOPS SDS Running Buffer along with a protein standard. The SDS-PAGE gels were subsequently stained with 1% Coomassie Brilliant Blue and de-stained overnight before imaging (Gauci et al., 2013).

#### 2.1.6 Amino acid sequence determination

Bands of interest were extracted from an SDS-PAGE gel and trypsin-digested. The digested protein samples were sequenced using a nanoLC MS/MS platform (Creative Proteomics; Shirley, NY). The peptide mapping data were used to construct a peptide sequence by combining the initial sequence data within the N-terminal cleavage window and C-terminal cleavage window, removing any sequence overlap. The final amino acid sequence was subsequently aligned using the NCBI BLASTp database (Altschul et al., 1990).

#### 2.1.7 Exoprotein analysis procedure

Each SDS-PAGE gel was analyzed for band intensity determination using ImageJ (Schneider et al., 2012), and the intensity of the image was set to 8-bit (black and white) and inverted. The intensity values and areas were measured for the bands of interest and were used to calculate the percent change from the control.

#### 2.1.8 Hemolysis assay

4EB treatments of 0.2, 0.4, 0.6, and 0.8 mg/mL were used. All samples were incubated at 37 °C, 200 rpm for 15 h. After incubation, the OD_600_ of each 4EB-treated culture was normalized to the untreated culture so that each treated and untreated culture had approximately the same number of cells (OD_600_ = 6.0 to 6.5). 5 mL of each culture was centrifuged at 4,000 x *g* for 20 min, 4 °C. The supernatant was removed and was sterilized using a 0.22 μm filter. The filtered supernatant was added (200 μL) into tubes containing nuclease-free water with 50 μL of 5% defibrinated sheep’s blood (Lampire, United States), and the samples were incubated at 37 °C for 1 h. Sterile LB broth served as the negative control and a sample containing 1% Triton X-100 (Research Products International) was used as the positive control. After the 1-h incubation, samples were centrifuged at 17,000 x *g* for 2 min and 150 μL of supernatant was transferred into polystyrene 96-well plates in triplicate. Hemolysis was analyzed at 540 nm using a Victor® 3V plate reader.

#### 2.1.9 Staphyloxanthin assay

Staphyloxanthin (STXN) production was analyzed as described (Vila et al., 2019). Treatments were prepared containing 0.2, 0.4, 0.6, and 0.8 mg/mL 4EB. The samples were incubated at 37 °C, 200 rpm for 24 h. After incubation, the OD_600_ of each 4EB-treated culture was normalized to the untreated culture so that each treated and untreated culture had approximately the same number of cells (OD_600_ = 6.0 to 6.5). The samples were then centrifuged at 4,000 x *g*, 4 °C for 20 min. The supernatants were removed, and the cell pellets were washed with 1x TBS (tris-buffered saline, pH 7.4–8.0) to remove the residual 4EB. The cell pellets were resuspended in 1 mL of 100% methanol (Fisher Scientific), thoroughly vortexed, and were incubated at 65 °C, 300 rpm for 5 min. The samples were subsequently centrifuged at 17,000 x *g* for 1 min, and the supernatants containing the pigment were transferred (150 μL) in triplicate to polystyrene 96-well plates. Methanol (100%) served as the negative control. STXN production was analyzed at 465 nm using a Victor® 3V plate reader.

#### 2.1.10 Total RNA extraction

Cultures were incubated at 37 °C, 200 rpm, for 15 h. The sample cultures were subsequently harvested at 3, 9, 15, and 24 h and were maintained on ice for 1 h to preserve the total RNA. The OD_600_ of each 4EB-treated culture was normalized to the untreated culture so that each treated and untreated culture had approximately the same number of cells (OD_600_ = 6.0 to 6.5). The cell samples were centrifuged, the supernatants decanted, and the pellets were maintained on ice for the remainder of the RNA extraction procedure.

Briefly, following a modified protocol for the RNeasy Mini Kit (QIAGEN), cells were lysed by bead-beating for 3 min using Lysing Matrix B tubes and Buffer RLT. Cell lysate was transferred into the RNeasy Spin Column, centrifuged, and washed according to the manufacturer’s instructions. Total RNA was eluted from the spin columns with nuclease-free water, and samples were stored at −80 °C. Two RNA extractions were performed for each biological replicate, and all timepoints were conducted in triplicate. RNA was subsequently treated with DNase 1 (Promega RQ1 RNase-free DNase) following the suggested protocol, and the DNase-treated samples were cleaned and concentrated (Monarch® RNA Cleanup Kit; NEB). Following a confirmation amplification of the RNA to confirm the absence of DNA, cDNA was synthesized (ProtoScript® First Strand cDNA Synthesis Kit; NEB) following the manufacturer’s recommended instructions with an RNase inhibitor added (Applied Biosystems). For each RNA sample, three cDNA syntheses were performed (plus the negative control reaction), and the three cDNA synthesis products were combined to create one cDNA pool per total RNA product. The cDNA was stored at −20 °C and was subsequently used for real-time quantitative PCR (RT-qPCR) analysis.

#### 2.1.11 Biofilm RNA extraction

Untreated and 4EB-treated cultures (150 μL) were pipetted into polystyrene 96-well plates, laboratory film-sealed and incubated with aeration at 37 °C, 200 rpm, for 15 h. The 96-well plates were harvested at 15 h and maintained on ice for 1 h to preserve the total RNA. The supernatants were separated from the biofilms and discarded. Using sterile RNase-free scraping tools and ice-cold DEPC-treated water, the biofilms were scraped from the polystyrene plates and transferred into sterile tubes. The harvested biofilm cells were vortexed, and the OD_600_ of each sample was measured. The OD_600_ of each 4EB-treated biofilm sample was normalized to the untreated biofilm sample. All samples were centrifuged at 17,000 x *g* for 2 min, the supernatants were discarded, and the biofilm pellets were maintained on ice. The remaining total RNA extraction procedure was completed as described above using the RNeasy Mini Kit (QIAGEN).

#### 2.1.12 Reverse transcription-quantitative PCR

For assessing *in vitro* gene expression, RT-qPCR was performed for the gene transcripts of interest relative to *16S rRNA* expression using a dye-based assay. Quantitative PCR was performed using the Applied Biosystems™ 7,500 Fast Real-Time PCR system with SYBR Select Master Mix (2x) (ROX dye). All samples were assayed in triplicate. The primer sequences were as follows: *16S rRNA* forward primer (1369) 5′- CGGTGAATACGTT-CYCGG −3′, reverse primer (1492) 5’-GGWTACCTTGTTACGACTT-3′ (Suzuki et al., 2000). All other primers are listed in Table 1. Cycle conditions were as follows: hold at 50 °C for 2 min, hold at 95 °C for 2 min, followed by 40 cycles of denaturation at 95 °C for 15 s and anneal/extend at 56 °C for 30 s. A standard curve and melt curve analysis were obtained in tandem for each assay. The relative expression of the 4EB-treated samples was determined using the Livak 2^-ΔΔCt^ method (Riedel et al., 2014).

**Table 1:** Primers for PCR and reverse-transcription quantitative PCR (RT-qPCR) analysis.

Primer name	Primer (5′ – 3′)	Source
agrA-RT-Fwd	TGATAATCCTTATGAGGTGCTTGA	Yehia et al. (2022)
agrA-RT-Rev	CACTGTGACTCGTAACGAAAATAAT	
ccpN1-RT-Fwd	TTGAGGTTAAGGACTATATG	This study
ccpN1-RT-Rev	GAACACACACCAACAAA	
ccpN2-RT-Fwd	TGACACGTATGCCTAATGTCACT	This study
ccpN2-RT-Rev	TTTCAATCATTCTATCTGCTGCGT	
crtM-RT-Fwd	TGATGACAGTATAGATGTTTATGG	Majerczyk et al. (2008)
crtM-RT-Rev	ACATGCTGAAGCGCCATCATG	
degA-RT-Fwd	CATCGCACAATACATTTCATCCC	This study
degA-RT-Rev	TCTGTATAGCCTTGATGGTCATTT	
emp-RT-Fwd	GGAAACATTGTGCCAGAG	This study
emp-RT-Rev	AGTGGTGGTTTACTTTGTTT	
entA-RT-Fwd	TGTACGAACAAGGTGGCGACAT	This study
entA-RT-Rev	GATAGTTTTATTATCAGCATAC	
entD-RT-Fwd	GCGTCTATATAATGCTAAGG	This study
entD-RT-Rev	CGAACATGTCATGAAATAAC	
graR-RT-Fwd	CGTCGTGTCTATGAGTTTAC	This study
graR-RT-Rev	AAATCGTCTGATCACCTTT	
graS-RT-Fwd	AACCATTACAGAATTTGTGC	This study
graS-RT-Rev	CCAGCATCGAGTTTATACG	
hla-RT-Fwd	TCTTGGAACCCGGTATATGG	Bulock et al. (2022)
hla-RT-Rev	AGCGAAGTCTGGTGAAAACC	
icaA-RT-Fwd	CTTGGATGCAGATACTATCG	Jafarı-sales and Pashazadeh (2020)
icaA-RT-Rev	GCGTTGCTTCCAAAGACCTC	
ilvD-RT-Fwd	GATGTCATTCACCCTCTTG	This study
ilvD-RT-Rev	CGCCGCCAACTTTAATA	
iolG-RT-Fwd	CATGATTGAAGCGGCTAATA	This study
iolG-RT-Rev	AAAGGTTTACCAACCACAC	
lukDv-RT-Fwd	GGTAGAGATAGTTATGACCCA	This study
lukDv-RT-Rev	AGGCATTTGATGTGTTGG	
lukEv-RT-Fwd	CACCTTTAGCATCTCCGATTCA	This study
lukEv-RT-Rev	CATTTCTTACTACTTACATCCTCCGTA	
lytM-RT-Fwd	ACTACACAGCGAGTCAAA	This study
lytM-RT-Rev	ATTACCATCATGGCTGTTATAC	
malL-RT-Fwd	GGTAATCCAGAAGTTAGAGATG	This study
malL-RT-Rev	TAAGTCACCCGCTTCAA	
malR-RT-Fwd	CACAAACGACAGTCTCAAATA	This study
malR-RT-Rev	GGCATGCTTTCATCAATTAAC	
oppB-RT-Fwd	TGCTGTATCTGTACCATCTT	This study
oppB-RT-Rev	GATAATGCAAGTGACGGTAATA	
RNAIII-RT-Fwd	ATCGACACAGTGAACAAATTCAC	Kim et al. (2021)
RNAIII-RT-Rev	CTCTACTAGCAAATGTTACTCAC	
saeR-RT-Fwd	CGCCTTAACTTTAGGTGCAGATGAC	Gao et al. (2020)
saeR-RT-Rev	ACGCATAGGGACTTCGTGACCATT	
saeS-RT-Fwd	TTATACCAGAACTACAAGAACG	This study
saeS-RT-Rev	ATCACTGCTTACACTGATTT	
splC-RT-Fwd	GGAATTCAGGATCACCAGTTCT	This study
splC-RT-Rev	GCGTAAAGTATACGGCACCA	
sspA-RT-Fwd	GTAGTTGTAGGTAAAGATACTC	Han et al. (2021)
sspA-RT-Rev	CCATTTGGATAATTGTCTTG	
treA-RT-Fwd	ATGACGGACCCACATTT	This study
treA-RT-Rev	GAATTGTCTCGTGACTTCTG	
treR-RT-Fwd	ACTGAAGTTGTTGTGAATGA	This study
treR-RT-Rev	CCGACGAGTTCTAACAATATG	
αPSM-whole-Fwd	ATGTCAAATTCATGAGC	This study
αPSM-whole-Rev	AACAAGACTCACCTCACATCAA	
psm-α1-Fwd	TAA TGG GTA TCA TCG CTG GC	This study
psm-α2-Rev	TACTTACCAGTGAATTTCTC	
psm-α3-Fwd	CATTCACATGGAATTCGTAG	This study
psm-α4-Rev	GTATTCAATTCGCTTAAATT	
psm-β1-Fwd	ATGACTGGACTAGCAGAAGC	This study
psm-β1-Rev	TAGAATCCAAATAATTTACC	
psm-β2-Fwd	CCTTCCATTGAAAACACTCCTTA	This study
psm-β2-Rev	TCAATATGTTATGTAAGTAATC	
psm-δ-Fwd	GTTGATGAGTTGTTTAATTT	This study
psm-δ-Rev	CTTATTTTTTAGTGAATTTG	
psm-δ-Fwd	GTTGATGAGTTGTTTAATTT	This study
psm-δ-Rev	CTTATTTTTTAGTGAATTTG	
walK-RT-Fwd	TACAATCCCTTCATACTAAACT	This study
walK-RT-Rev	GTGCATTTACGGAGCCCTT	
walR-RT-Fwd	GAAGAACCAGACATCGTATTA	This study
walR-RT-Rev	TCTGAATCTTTAGCAGTAAGC	
yqfL-RT-Fwd	CGTGCTTCTGTTGCATATC	This study
yqfL-RT-Rev	CCAAAGAAATGTATCGCACTTA	

#### 2.1.13 Resazurin assay

The metabolic activity of *S. aureus* ATCC 6538 was analyzed using the resazurin assay as described (Muniyasamy and Manjubala, 2024; Chung et al., 2021) with modifications. A 1 mg/mL aqueous resazurin stock solution was prepared in advance as described (Oeschger and Erickson, 2021), and the stock solution was stored at −20 °C. Prior to the start of the assay, the 1 mg/mL stock solution was used to prepare a 0.01% (v/v) working stock using sterile 1x PBS (phosphate buffer saline). Cultures were incubated at 37 °C, 200 rpm, for 3, 6, 15, and 24 h. At each timepoint, 100 μL of the cells were immediately transferred into polystyrene (white, opaque) 96-well plates in triplicate. 60 μL of 0.01% resazurin was pipetted into each well, and the microtiter plates were incubated in the dark at 37 °C, 200 rpm, for 3 h. Sterile LB broth was used as the negative control. The resorufin fluorescence was analyzed at 540/590 nm (excitation/emission) using a SpectraMax® iD5 Multi-Mode Microplate Reader (Molecular Devices). The resazurin assay was repeated for a total of three biological replicates.

### 2.2 *In silico* assays

#### 2.2.1 Phenol-soluble modulin identification

Phenol-soluble modulin (PSM) genes were identified within the *S. aureus* ATCC 6538 genome^1^ using the PSM peptide sequences listed in Schwartz et al. (2012). The α1-4 *psm* and β1-2 *psm* genomic regions and the *δ*-toxin (*hld*) gene were PCR-amplified using primers listed in Table 1. PCR amplification was carried out with DreamTaq Green PCR Mastermix (2X) (Thermo Scientific™) following the manufacturer’s instructions, and the amplicons were Sanger-sequenced (Georgia State University, United States). The *psm* gene sequences were reverse translated^2^ and these predicted peptide sequences were aligned with the peptide sequences published in Schwartz *et al*., 2012 for gene confirmation. All putative *psm* genes were PCR-amplified and sequenced for confirmation (Supplementary Table 1). Due to each α*psm* gene being 63–69 nucleotides in length, the α1/α2 and α3/α4*psm* genes were transcriptionally analyzed in combination.

#### 2.2.2 Molecular docking analysis

One hundred proteins consisting of 75 transcriptional regulators and 25 virulence-associated enzymes were identified within the *S. aureus* ATCC 6538 genome (Makarova et al., 2017). The crystal structures of proteins were obtained from the Protein Data Bank (Berman et al., 2000) and predicted protein structures were obtained from AlphaFold (Jumper et al., 2021). The protein structure identifications are listed in Table 2.

**Table 2:** Protein structure identifications used in this work.

Protein	Identification	Protein	Identification
AdhR	A0A0D6H7Y4	LrgB	T1Y6S2
AgrA	PDB 4XYO	LytM	A0A846KJR3
AgrB	A0A6M1X709	LytR	PDB 6M8O
AgrC	PDB 4BXI	MalR	PDB 3HCW
AgrD	O33589	ManR	A0A831E4Y1
ArcR	A0A0E1XAT8	MarR	D2J5X2
ArlR	PDB 6IJU	MepR	PDB 4L9J
ArlS	A0A0E1X7D0	MgrA	PDB 2BV6
Atl	P0C5Z8	MgsR	A0A811GP77
CcpA	PDB 7E5W	MhqR	A0A6N3DGZ3
CdaA	A0A898CM21	MntR	A0A2X2JVB9
CdaR	A0A413EDR1	MraZ	A0A0E0VNA0
CggR	A0A2K4DFH5	MsrR	A0A7U7IEH0
CidA	A0A0E1XCT9	NorG	A0A806L248
CidB	A0A7U7F143	NreA	PDB 6IZJ
ClfA	A0A173GQ76	NreC	A0A0E1VY59
CodY	PDB 5EY0	Nuc	PDB 2EYH
CtrN	A0A0E1VPT9	PhoP	A0A0E0VRI0
CrtP	A0A2S6DFP9	RecX	A0A0E0VQ39
CrtQ	A0A2S6DFN7	Rot	PDB 4RBR
CtsR	A0A806KZL7	RsbU	A0A6D2H1N8
CymR	PDB 3T8R	RsbV	A0A0H2XG98
CynR	W8TU56	RsbW	A0A806KUQ6
DegA	A0A0D3Q452	SaeR	PDB 4QWQ
DesR	PDB 4LE2	SaeS	Q2YSM6
EzrA	PDB 4UXV	SarA	PDB 2FRH
FnbA	X2EXF7	SarR	A0A7U4AVH7
GabR	A0A229LWF0	SarS	PDB 1P4X
GlnR	PDB 7TEA	SarU	A0A0B6XSX0
GlpP	A0A7U7IEE4	SarX	PDB 5HS5
GlvR	A0A511H269	SdrC	PDB 6LXH
GraS	A0A806L004	SdrE	A0A811EBH2
GraR	A0A0E1VU17	SigB	A0A5C8X738
HisZ	A0A0E1XAP7	SlyA	A0A844HCK6
Hfq	A0A806L3Z8	SpxA	A0A806KNF4
HssR	A0A806KWL8	SrrA	H9BRN8
IcaA	A0A7U7F068	SspA	A0A7H2KDK6
IcaB	A0A830YSS5	TcaR	PDB 3KP7
IcaC	A0A380DPQ8	TreR	A0A0E1XBQ0
IcaD	W8U2R0	Ung	A0A0E1VJL6
IcaR	PDB 3GEU	VraR	PDB 4GVP
KatA	Q9L4S1	WalK	A0A0H3K0H8
KdpE	A0A380EKJ8	WalR	A0A1L6C153
KPFDPMNM_01268	Q2G0D0	YbbH	W8UU09
KPFDPMNM_01724	A0A0E1XA44	YdjF	A0A0H2XH29
KPFDPMNM_02241	Q6G6V7	YodB	W8UVY3
LiaR	W8TS97	YqfL	A0A811EBI0
LrgA	A0A7U4ERP3	YydK	A0A3A3ALG4

The identifications beginning with “PDB” were obtained from Protein Data Bank. All other proteins were obtained from AlphaFold.

Molecular docking between 4EB and the proteins of interest was quantified using AutoDock Vina v1.1.2 (Trott and Olson, 2010) and Open Babel v3.0.1 software (O'Boyle et al., 2011). Water molecules and any additional ligands were removed using AutoDockTools (Huey et al., 2012). For AutoDock Vina, the exhaustiveness was set to 20 for thoroughness of search and accuracy, and all other default parameters were used. For each protein, the form of the grid box was defined as a hexahedron that covered the entire protein domain, allowing for 4EB to bind in several conformations. All 100 molecular docking experiments were replicated three times, with eight of the best-fit ligand poses being generated during each experimental replication. The ligand poses with the most negative ΔG values (strongest binding affinity) were averaged to determine the mean ΔG values of the three docking experiments. Structural images of the protein-ligand binding sites were generated using BIOVIA (Visualizer BDS, 2024) for 2D imaging and PyMOL v3.0.2 (DeLano, 2002) for 3D imaging. The experimental process of molecular docking was carried out using a scripting workflow.

#### 2.2.3 Phylogenetic analysis

Phylogenetic analysis was performed for the 100 proteins of interest. Protein PDB files (Table 2) were aligned using MUSCLE Multiple Sequence Alignment (Edgar, 2004). The alignment was transformed into a phylogenetic tree and was edited and annotated using TreeViewer software (Bianchini and Sánchez-Baracaldo, 2024). The CLUSTAL multiple sequence alignment by MUSCLE (FASTA format) was added onto the phylogenetic tree for alignment visualization.

For conserved domain identification, proteins with the most favorable binding to 4EB were analyzed using the NCBI Conserved Domain Database (Yang et al., 2020). Known protein DNA-binding sites and known active sites were located using Protein Data Bank (Berman et al., 2000), AlphaFold (Jumper et al., 2021), and the NCBI Conserved Domain Database.

#### 2.2.4 Statistical analysis

Data were analyzed for statistical significance using Student’s *t*-test. All experiments were conducted with a minimum of three independent biological replicates. *p* < 0.05 was considered significant.

## 3 Results

### 3.1 Effect of 4EB on *Staphylococcus aureus* growth and metabolic activity

At a concentration of 0.8 mg/mL, 4EB attenuated planktonic growth by 47 ± 7% after 24 h of growth (MIC_50_; Kowalska-Krochmal and Dudek-Wicher, 2021; Supplementary Figure 1) and reduced biofilm formation by 88% (Campbell et al., 2020). The corresponding metabolic activity was analyzed using resazurin, a viability and activity indicator. During treatment of planktonically grown cells with 0.8 mg/mL 4EB, *S. aureus* metabolism was reduced 0–18% over 24 h compared to an untreated control (Supplementary Figure 2).

### 3.2 4EB impacts *Staphylococcus aureus* 3D biofilm structure

**Figure 1:**
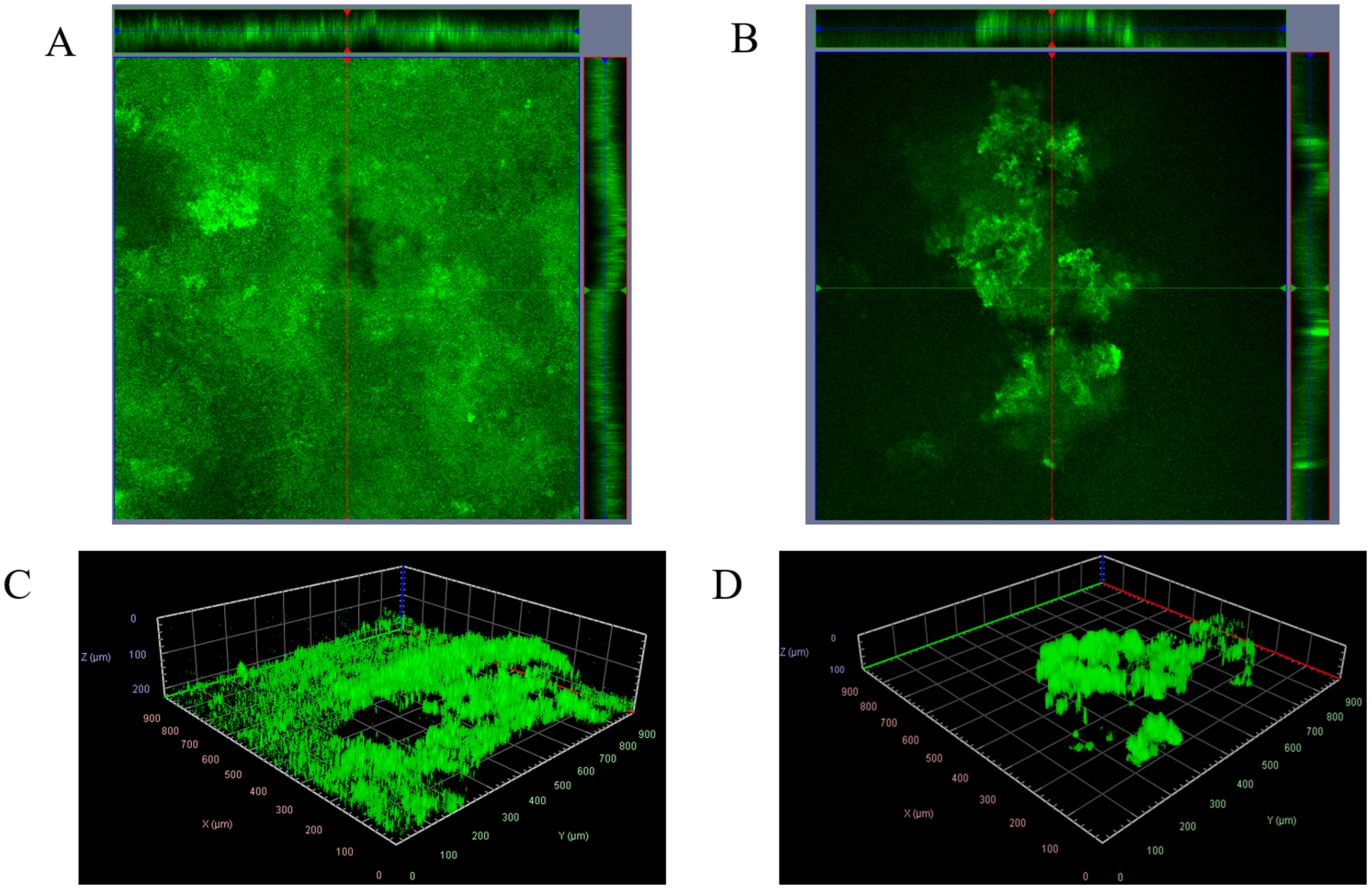
3D biofilm structures after 24 h of growth. Representative orthogonal views of the untreated control **(A)** and 0.8 mg/mL 4EB **(B)**. Corresponding 3D views for the representative images with a 35% threshold value to remove noise for the untreated control **(C)** and 0.8 mg/mL 4EB **(D)**. Cells were stained with 100 ppm acridine orange before imaging on an LSM510 (Zeiss). Images were visualized using Zeiss Zen Lite software.

Biofilms treated with 0.8 mg/mL 4EB prior to inoculation formed characteristic tower structures after 24 h but lacked the extensive surface area coverage seen in untreated control biofilms (Figures 1A,B). The maximum height of the 4EB-treated biofilms was significantly less than the untreated controls (control: 131 ± 5 μm, 4EB-treated: 88 ± 3 μm; *p* < 0.03). Tower structures in the 4EB-treated biofilms appeared “patchy” relative to the more uniform and bulbous structures seen in the controls (Figures 1C,D). The mean fluorescent intensity for each biofilm is indicative of the amount of biomass that is present. A comparison of 4EB-treated biofilms versus untreated control biofilms determined a 47 percent greater average fluorescence intensity in the control biofilms (1.77 ± 0.09 × 104 RFU) compared to the 4EB-treated biofilms (1.20 ± 0.03 × 104 RFU). These data indicated that 4EB treatment reduced biofilm biomass accumulation (*p* < 0.04). Together these quantitative measurements demonstrated that 4EB treatment impacted the three-dimensional biofilm structure.

### 3.3 4EB inhibits *Staphylococcus aureus* alpha-hemolysin and serine protease production

**Figure 2:**
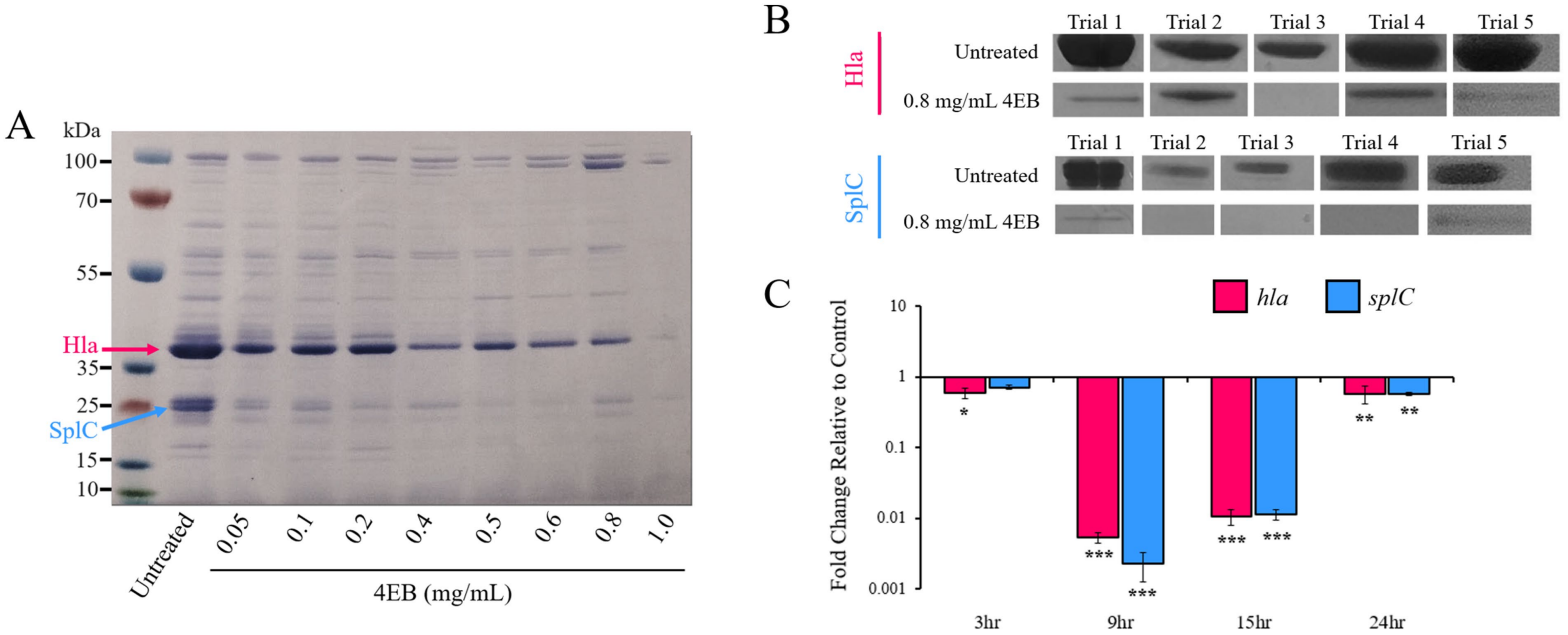
*Staphylococcus aureus* Hla and SplC exoprotein production during 4EB treatment. **(A)** Exoprotein production during treatment with various concentrations of 4EB at 15 h of growth using normalized cell amounts. **(B)** The intensity (%) of exoprotein production was quantified for five separate experiments (trials 1–5; shown) during treatment with 0.8 mg/mL 4EB. **(C)** Relative expression of *hla* and *splC* genes during treatment with 0.8 mg/mL 4EB. The results represent the mean ± SEM of the triplicate RT-qPCR determinations of each cDNA sample obtained from three replicate planktonic cultures. Statistical significance: *, *p* < 0.05; **, *p* < 0.01; ***, *p* < 0.001.

We evaluated extracellular protein production in response to 4EB treatment. Treated samples and untreated controls were normalized to the same cell concentration prior to protein extraction (OD_600_ = 6.0 to 6.5). Concentrations of 4EB ranging from 0.05 to 1.0 mg/mL were selected and the exoproteins were profiled by SDS-PAGE (Figure 2A). Exoprotein production decreased with increasing 4EB concentration. Two prominent protein bands, indicated by the blue and pink arrows, had the greatest decrease in expression. These bands were extracted and sequenced. The protein band at ~35 kDa was identified to be alpha-hemolysin (Hla, 86.7% identity), and the protein band at ~20 kDa was identified to be serine protease C (SplC, 90.6% identity) (Supplementary Figures 3, 4). Exoprotein production was measured for cells grown for 15 h with 0.8 mg/mL for five trials (Figure 2B). The average Hla and SplC production were significantly reduced relative to untreated controls by 64 ± 16% (*p* < 0.001) and 56 ± 9% (*p* < 0.001), respectively, following 4EB treatment.

Reverse-transcription quantitative PCR (RT-qPCR) analysis was performed to determine the effect of 4EB on *hla* and *splC* expression. Transcript levels were quantified at 3, 9, 15 and 24 h of growth with 0.8 mg/mL 4EB. Transcription of *hla* and *splC* was most significantly downregulated (~100 fold or more) at 9 h and 15 h (early- and mid-stationary phases of growth) compared to the untreated control (Figure 2C).

**Figure 3:**
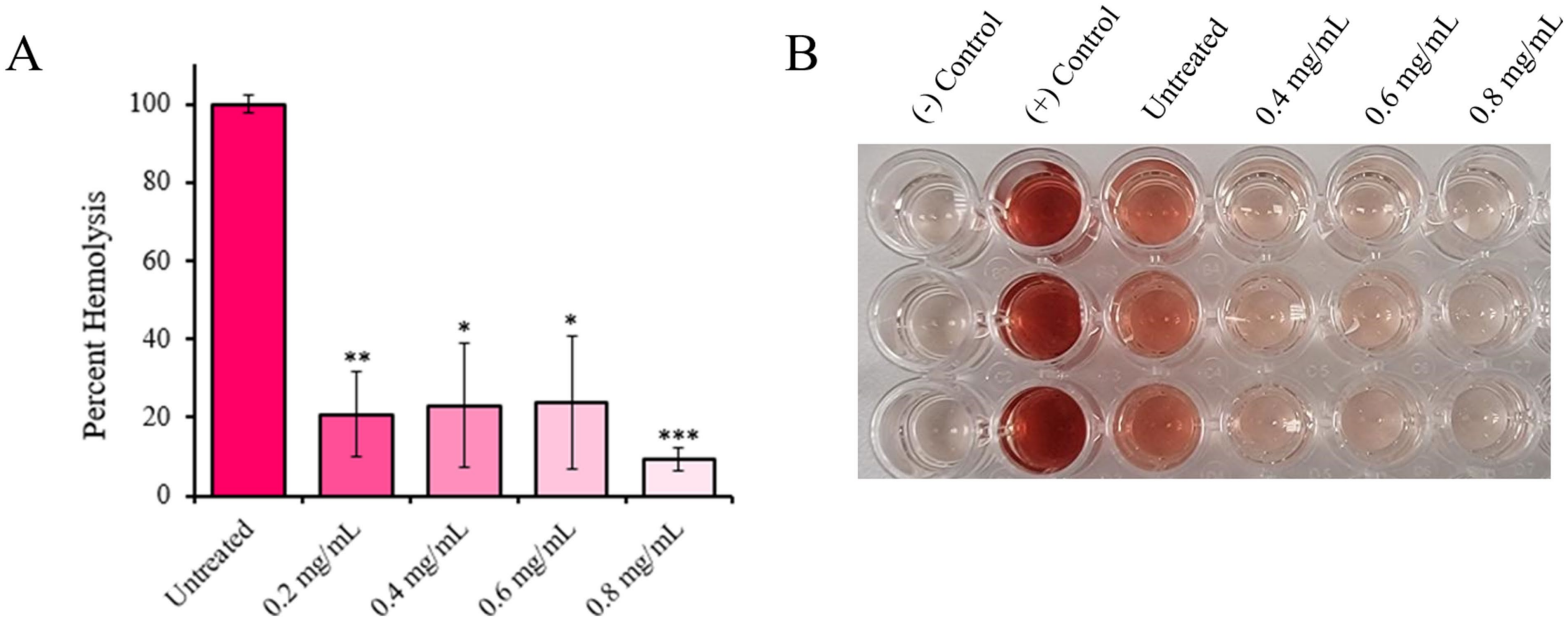
Hemolysis during 4EB treatment. *S. aureus* alpha-hemolysis was measured at 15 h of growth, and the percentage of hemolysis relative to the untreated control was measured during treatment with 0.8 mg/mL 4EB **(A)** Percentages of hemolysis are relative to the untreated control **(B)** Hemolysis supernatants; the negative control was sterile LB broth, and the positive control was 1% Triton X-100. Statistical significance: *, *p* < 0.05; **, *p* < 0.01; ***, *p* < 0.001.

Upon observing that Hla exoprotein production was significantly reduced by 4EB treatment, hemolysis activity was measured (at 15 h when Hla production levels were significantly inhibited; Figure 2B). Alpha-hemolysis was reduced by up to 87 ± 14% (*p* < 0.001) during treatment with 0.8 mg/mL 4EB (Figures 3A,B).

### 3.4 4EB affects additional *Staphylococcus aureus* virulence factors

**Figure 4:**
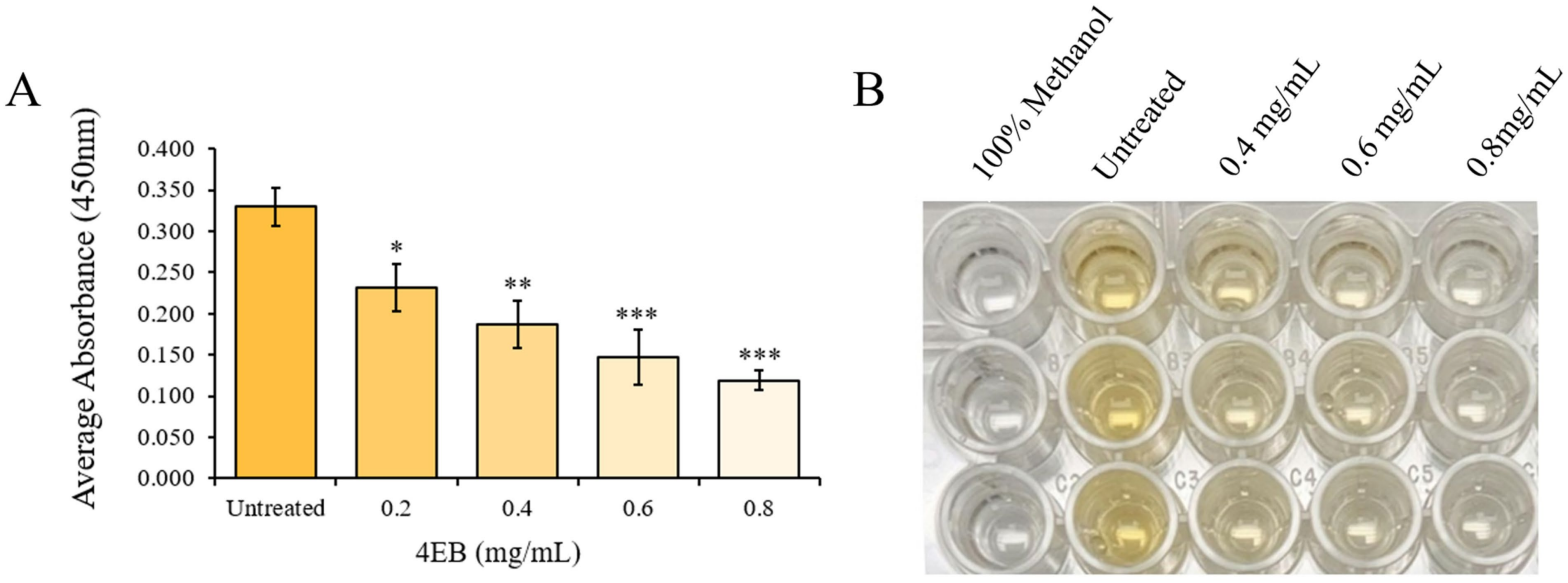
STXN production during treatment with 4EB. **(A,B)** STXN pigmentation production was measured during treatment with various concentrations of 4EB. Statistical significance: *, *p* < 0.05; **, *p* < 0.01; ***, *p* < 0.001.

We investigated whether additional virulence factors were affected by 4EB treatment. We visually observed that staphyloxanthin (STXN) pigmentation decreased when the cells were treated with 4EB. To test the impact of 4EB on STXN production, methanol extractions were performed followed by spectrophotometric analysis. During treatment with 4EB, STXN was decreased by up to 73 ± 7% (*p* < 0.001) following treatment with 0.8 mg/mL 4EB (Figures 4A,B).

**Figure 5:**
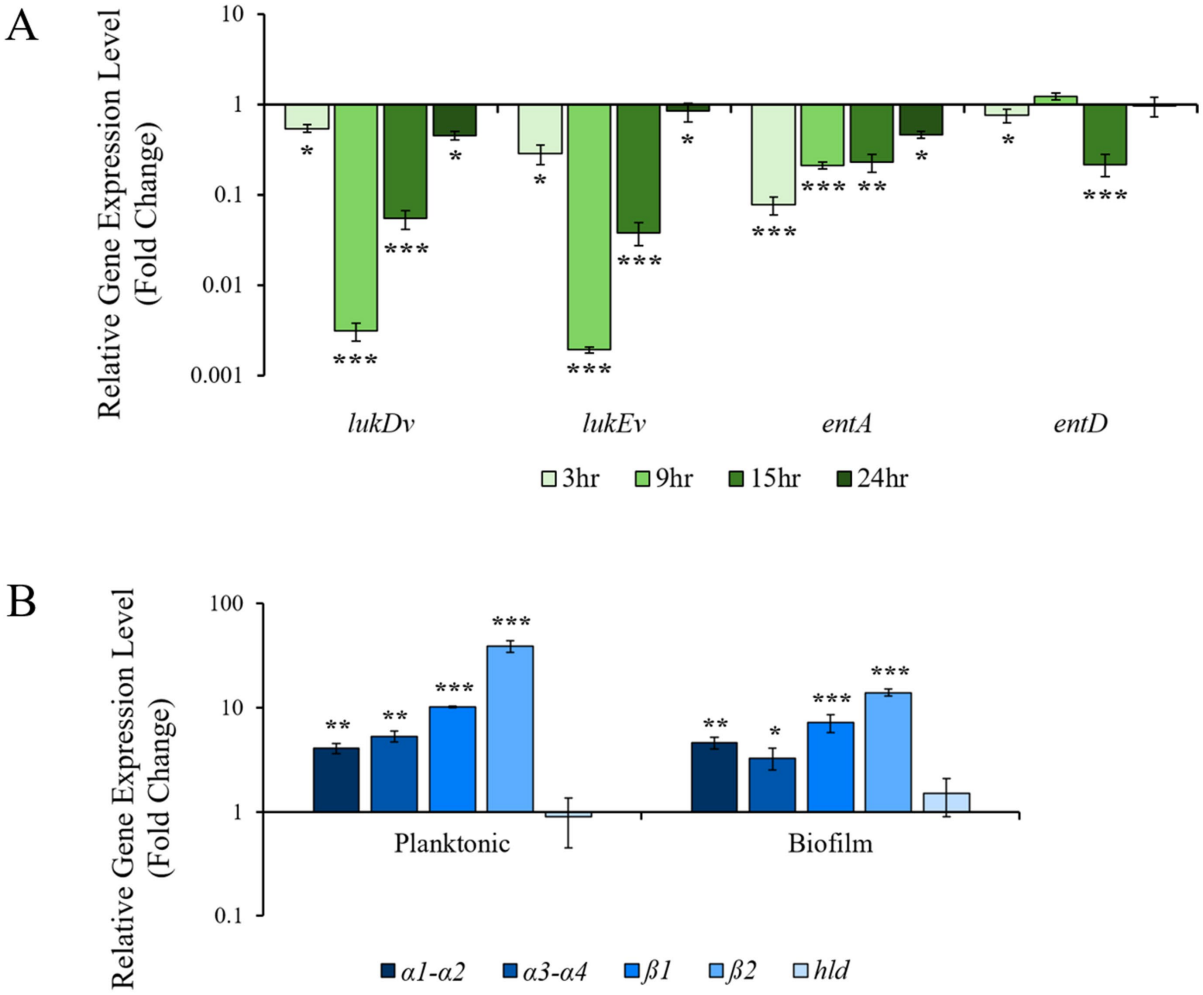
Relative expression levels for genes associated with *S. aureus* toxins during treatment of 0.8 mg/mL 4EB. The fold change shown is relative to the transcript levels in untreated control. **(A)** Leukocidins (*lukDv* and *lukEv*) and enterotoxins (*entA* and *entD*) were analyzed. **(B)** The phenol-soluble modulins (α*psm*, β*psm*, and *δ*-toxin) genes were analyzed. The results represent the mean ± SEM of the triplicate RT-qPCR determinations of each cDNA sample obtained from three replicate planktonic cultures. Statistical significance: *, *p* < 0.05; **, *p* < 0.01; ***, *p* < 0.001.

In *S. aureus*, three categories of toxins are produced that eliminate neutrophils and leukocytes: alpha-toxin (alpha-hemolysin), leukocidins, and phenol-soluble modulins (PSMs). We observed that 4EB decreased alpha-hemolysin production and alpha-hemolysis. Therefore, we also measured the effect of 4EB on leukocidin and PSM gene transcription. Strain ATCC 6538 does not contain *lukA* or *lukB* leukocidin genes, so only transcript levels for *lukEv* and *lukDv* leukocidins were quantified. Following 0.8 mg/mL 4EB treatment, *lukEv* and *lukDv* were both significantly downregulated at all timepoints and were reduced by 500-fold and 40-fold at both the 9 and 15 h times, respectively (Figure 5A). Two enterotoxins encoded by *entA* and *entD* are present within the ATCC 6538 genome, and their transcript levels were quantified. Transcription of *entA* was downregulated by 17-fold at 3 h. However, levels of transcripts for *entD* were variable with 4EB treatment, although they were downregulated 22-fold at 15 h. These results indicated that 4EB treatment significantly downregulated the expression of leukocidins and enterotoxins.

PSM toxin expression in response to 4EB treatment was investigated by quantifying gene expression levels. There are three classes of PSMs: αPSMs (α1, α2, α3, α4*psm*), βPSMs (β1, β2*psm*), and *δ*-toxin (*hld*). Using published PSM amino acid sequences (Schwartz et al., 2012), all *psm* genes were identified within the strain ATCC 6538 genome. The *psm* genes and *hld* are only expressed during the post-exponential growth phase (Wang et al., 2023); therefore, transcript levels for psm genes were quantified at the 15 h timepoint only. PSMs have been shown to have variable levels of expression during biofilm formation, when compared to planktonic growth (Wang et al., 2023), and so *psm* expression levels were quantified for both planktonic and biofilm modes of growth (Figure 5B). All *psm* genes were similarly expressed in both the planktonic and biofilm cultures treated with 4EB (Figure 5B). All *psm* genes were significantly upregulated following 4EB treatment, except for *hld* (δ-toxin), which was unaffected by 4EB treatment.

**Figure 6:**
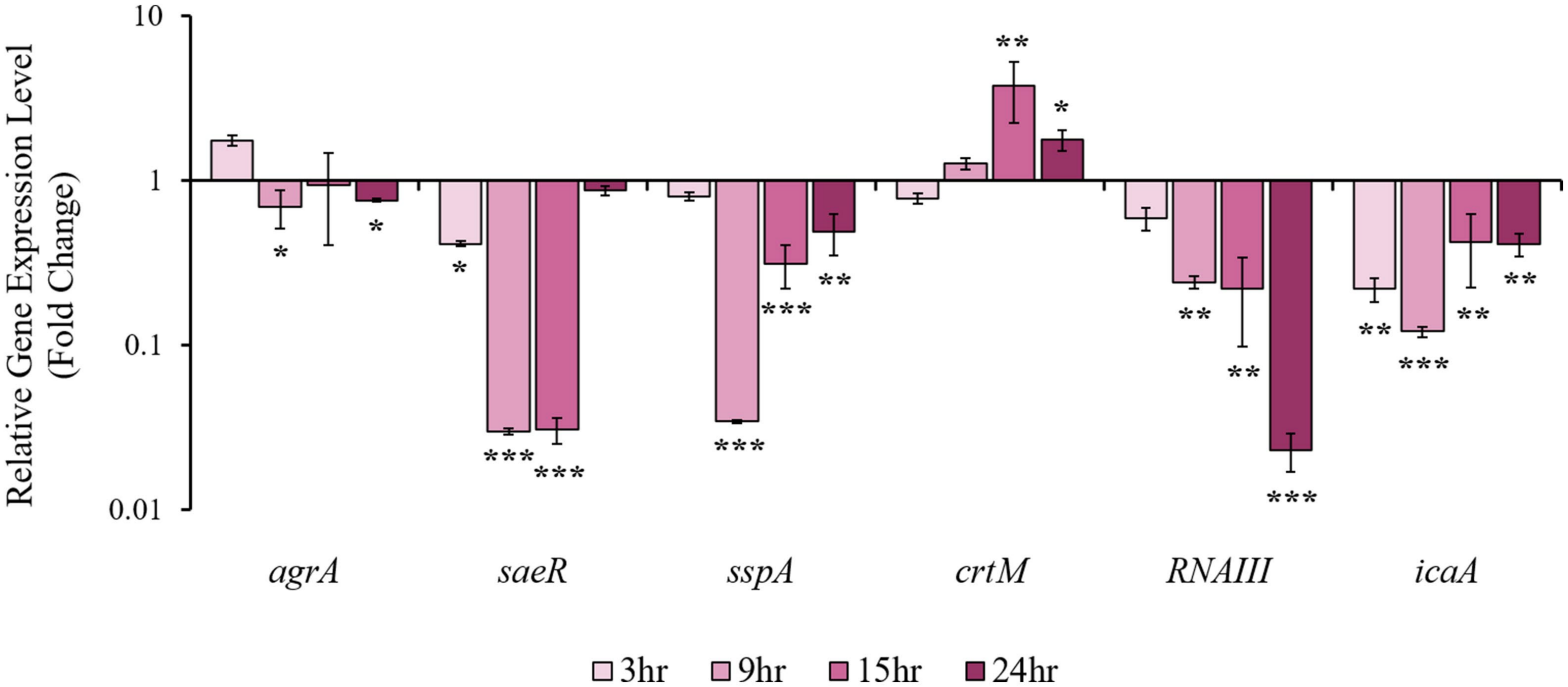
Relative expression levels for *S. aureus* virulence-associated genes. Transcript levels for regulatory-, biofilm-, and virulence-associated genes were quantified after 0.8 mg/mL 4EB treatment. The results represent the mean ± SEM of the triplicate RT-qPCR determinations of each cDNA sample obtained from three replicate planktonic cultures. Statistical significance: *, *p* < 0.05; **, *p* < 0.01; ***, *p* < 0.001.

To identify possible mechanisms of 4EB activity, the levels of transcripts for the following genes were measured: accessory gene regulator A (*agrA*), *sae* response regulator (*saeR*), V8 serine protease (*sspA*), dehydrosqualene synthase (*crtM*; STXN synthesis), *RNAIII*, and intracellular adhesion gene A (*icaA*) (Figure 6). All the genes tested were downregulated in response to 4EB treatment except for *crtM*, which was significantly upregulated at 9 and 15 h. Apart from *agrA*, transcript levels for all other genes changed from one- to two-log units over the sampling period.

We observed a change in the expression of select biofilm and virulence-associated genes by 3 h after treatment with 0.8 mg/mL 4EB (Figures 5, 6). To understand the relationship between 4EB concentration and its influence on early-stage gene expression, we investigated the expression of select biofilm and virulence-associated genes at two sub-MIC concentrations: 0.4 mg/mL and 0.8 mg/mL, and at two early-stage timepoints, 3 h and 9 h of growth. At 3 h, there was a significant difference in the level of gene expression for all of the evaluated genes, with the absolute expression level (i.e., either upregulation or downregulation) at 0.8 mg/mL greater than at 0.4 mg/mL (Supplementary Figure 5A). At 9 h, there was no significant difference in gene expression for any tested gene at 0.4 mg/mL versus 0.8 mg/mL (Supplementary Figure 5B).

### 3.5 Molecular docking analysis of 4EB interactions with transcriptional regulators

We tested the hypothesis that 4EB interacts with transcriptional regulatory proteins using *in silico* molecular docking. One hundred proteins within the ATCC 6538 genome were selected using the Protein Data Bank (Berman et al., 2000) and AlphaFold (Jumper et al., 2021); 75 proteins were transcriptional regulators, and 25 proteins were other virulence-associated enzymes (Supplementary Table 2). We docked the 100 proteins with 4EB using AutoDock Vina v1.1.2 (Trott and Olson, 2010). 27 proteins had binding affinities (ΔG values) between −5.6 and −5.9 kcal/mol, and 14 proteins had ΔG values more negative than −6.0 kcal/mol, potentially indicating a specific association (Table 3). All ΔG values for each protein-4EB interaction were chosen for the pose with the most favorable binding complex.

**Table 3:** *In silico* docking results for proteins of interest with 4EB.

**Regulatory proteins**		
Protein name	Average ΔG binding affinity (kcal/mol)	Protein function
MalR	−6.82 ± 0.32	HTH-type transcriptional regulator; maltose metabolism
TreR	−6.77 ± 0.06	HTH-type transcriptional regulator; trehalose metabolism
WalK	−6.40 ± 0.95	Sensor protein kinase
IcaR	−6.15 ± 0.35	HTH-type negative transcriptional regulator; biofilm operon *icaADBC*
YqfL	−6.20 ± 0.00	Putative pyruvate, phosphate dikinase regulator
KPFDPMNM_ 01724	−6.10 ± 0.00	Putative response regulatory protein
CodY	−6.03 ± 0.06	GTP-sensing transcriptional pleiotropic repressor
DegA	−6.03 ± 0.06	HTH-type transcriptional regulator
GraS	−6.03 ± 0.55	Sensor histidine kinase
SaeS	−6.03 ± 0.67	Histidine protein kinase
ArlS	−5.93 ± 0.64	Signal transduction histidine-protein kinase
CcpA	−5.85 ± 0.01	Catabolite control protein
CynR	−5.90 ± 0.26	HTH-type transcriptional regulator
SdrC	−5.87 ± 0.06	Serine-aspartate repeat-containing protein C
TcaR	−5.75 ± 0.06	HTH-type regulator
MsrR	−5.73 ± 0.06	Regulatory protein; cell wall associated
RsbU	−5.70 ± 0.17	Phosphoserine phosphatase
NreC	−5.63 ± 0.06	Oxygen regulatory protein
SarA	−5.63 ± 0.10	Regulatory protein; virulence genes regulator
HisZ	−5.60 ± 0.00	ATP phosphoribosyltransferase regulatory subunit
**Other proteins/Enzymes**		
KatA	−6.90 ± 0.00	Catalase
CrtN	−6.63 ± 0.35	Dehydrosqualene desaturase; Staphyloxanthin synthesis
Nuc	−6.28 ± 0.06	Nuclease
CrtP	−6.07 ± 0.75	Diapolycopene oxygenase; Staphyloxanthin synthesis
IcaC	−5.90 ± 0.00	putative poly-beta-1,6-N-acetyl-D-glucosamine export protein
IcaA	−5.80 ± 0.00	Poly-beta-1,6-N-acetyl-D-glucosamine synthase
CrtQ	−5.63 ± 0.06	4,4′-diaponeurosporenoate glycosyltransferase; Staphyloxanthin synthesis

Docking was measured in three independent experiments (mean ± s.d.). See Methods for details.

**Figure 7:**
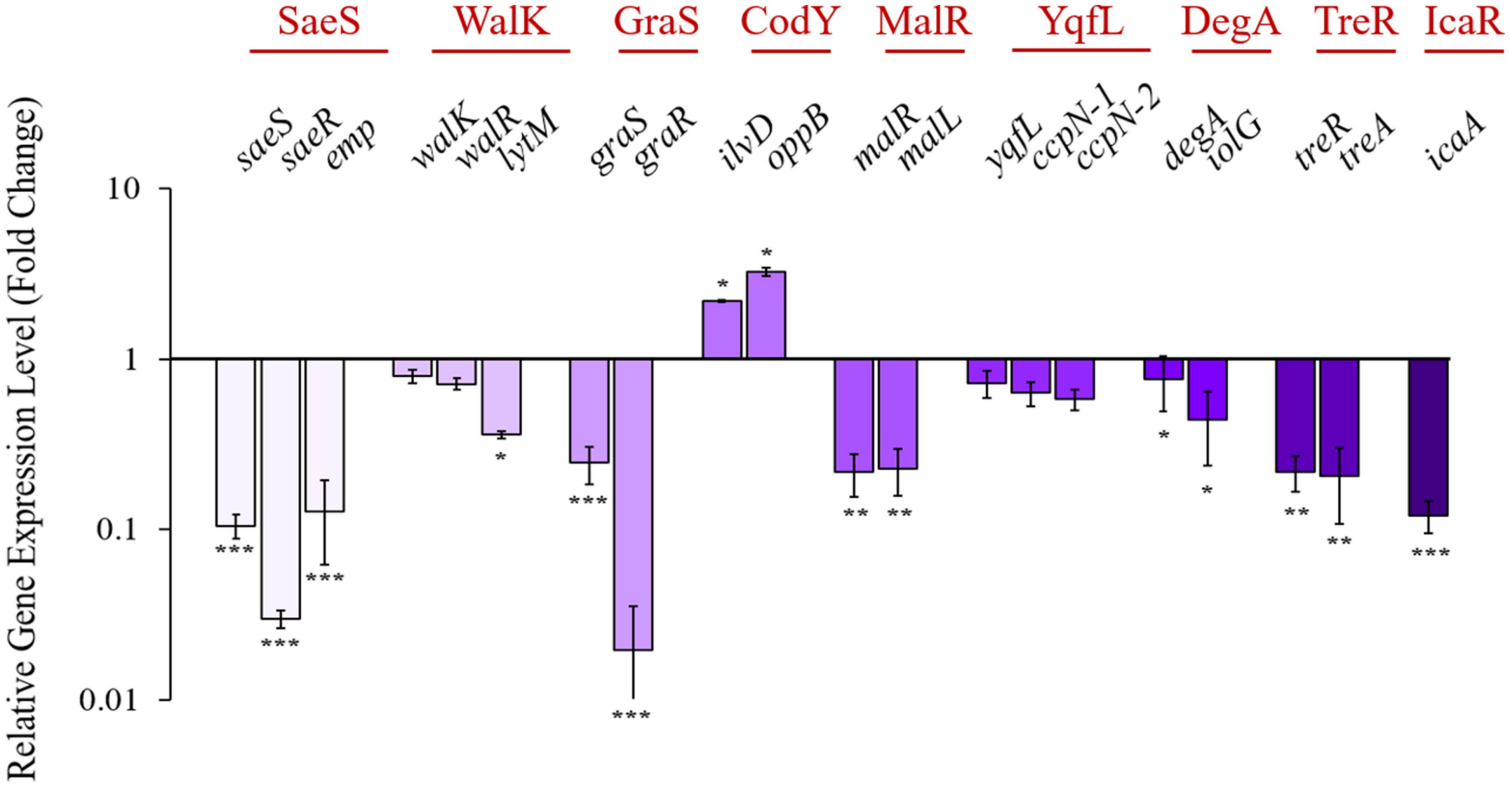
Relative expression levels for select *S. aureus* genes identified by in silico molecular docking to bind with high affinity to 4EB. Transcript levels for downstream regulons of the proteins of interest (red, top of figure) were quantified after 0.8 mg/mL 4EB treatment at 9 h. The results represent the mean ± SEM of the triplicate RT-qPCR determinations of each cDNA sample obtained from three replicate planktonic cultures. Statistical significance: *, *p* < 0.05; **, *p* < 0.01; ***, *p* < 0.001.

The validity of the 4EB-protein associations for the nine identified transcriptional regulators with ΔG values more negative than −6.0 kcal/mol (Table 3) were evaluated *in vitro* using RT-qPCR analysis. For each regulator, the expression of genes known to be under their control (Supplementary Table 3) was measured after 9 h of growth (late exponential/early stationary phase). For seven of the nine transcription regulators, 4EB caused a significant change in their transcription relative to the untreated control (Figure 7). The extent of the change varied from 2-fold to more than 80-fold at the measured timepoint.

**Figure 8:**
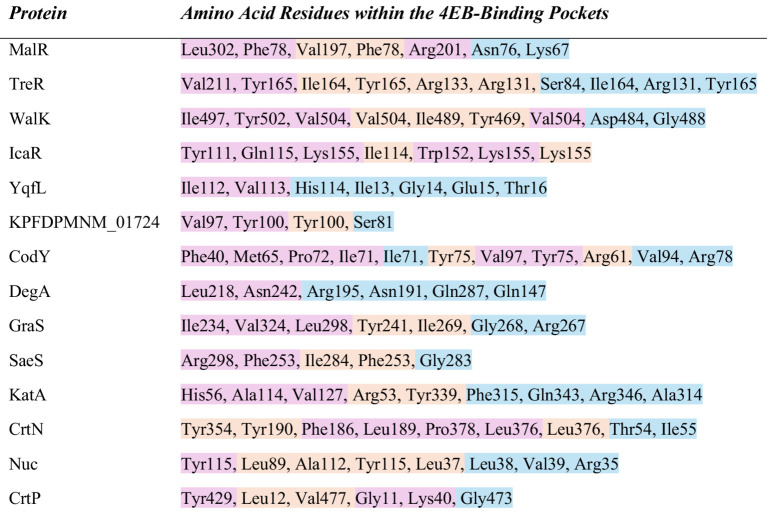
Amino acid residues that were found within the 4EB-binding pockets. Residues in pink represent binding with the alkoxy side chain of 4EB. Residues in orange represent binding with the phenyl ring of 4EB. Residues in blue represent binding with the carboxylic acid side chain of 4EB. Each of the proteins in the table associated with 4EB with a binding affinity (ΔG) less than −6.0 kcal/mol.

The 4EB-amino acid residue interactions for each of the 27 proteins were analyzed using the optimal binding confirmation of each complex (Supplementary Figure 6). The alkoxy moiety of 4EB formed alkyl bonds with hydrophobic amino acids during 84% of the interactions, with valine binding most frequently (Figure 8). The phenyl ring of 4EB associated with hydrophobic amino acids forming a pi-alkyl bond 83% of the time, with valine binding most frequently. The phenyl ring also formed pi-pi stacked bonds involving only tyrosine and phenylalanine residues. The carboxy moiety of 4EB formed conventional hydrogen bonds with several amino acids, most frequently arginine and glycine. 4EB-residue interactions for each of the 14 proteins with 4EB-binding complexes of ΔG < −6.0 kcal/mol are listed in Figure 8.

To understand the effect of the 4EB-residue interactions within each protein, we identified known DNA-binding sites, ligand binding sites, and active sites of the proteins listed within Figure 8 (ΔG < −6.0 kcal/mol) using the UniProt database (UniProt Consortium, 2015). All residues involved with binding 4EB (Figure 8) were cross-referenced to known binding sites (Supplementary Table 4). Proteins MalR, TreR, IcaR, CodY, and DegA are DNA-binding transcriptional regulators with the helix-turn-helix (HTH) DNA-binding motif (Table 3). The *in silico* analysis demonstrated that 4EB did not interact within the HTH region of these proteins (Figure 8; Supplementary Table 4). Proteins WalK, GraS, and SaeS are sensor kinase proteins, the essential phosphorylation component of two-component regulatory systems (TCSs). The *in silico* data determined that 4EB interacts with the histidine kinase domain of WalK, GraS, and SaeS (Figure 8; Supplementary Table 4). 4EB did not interact with the transmembrane domains or ligand binding sites of the sensor protein kinases (Supplementary Table 4). The sensor protein kinase SaeS was selected as a representative example for visualizing 4EB binding within the histidine kinase domain (Figure 10A). The transcriptional regulator CodY was selected as a representative example for visualizing 4EB’s interaction outside of the DNA-binding HTH-motif (ΔG = −6.03 kcal/mol) (Figure 10B).

**Figure 9:**
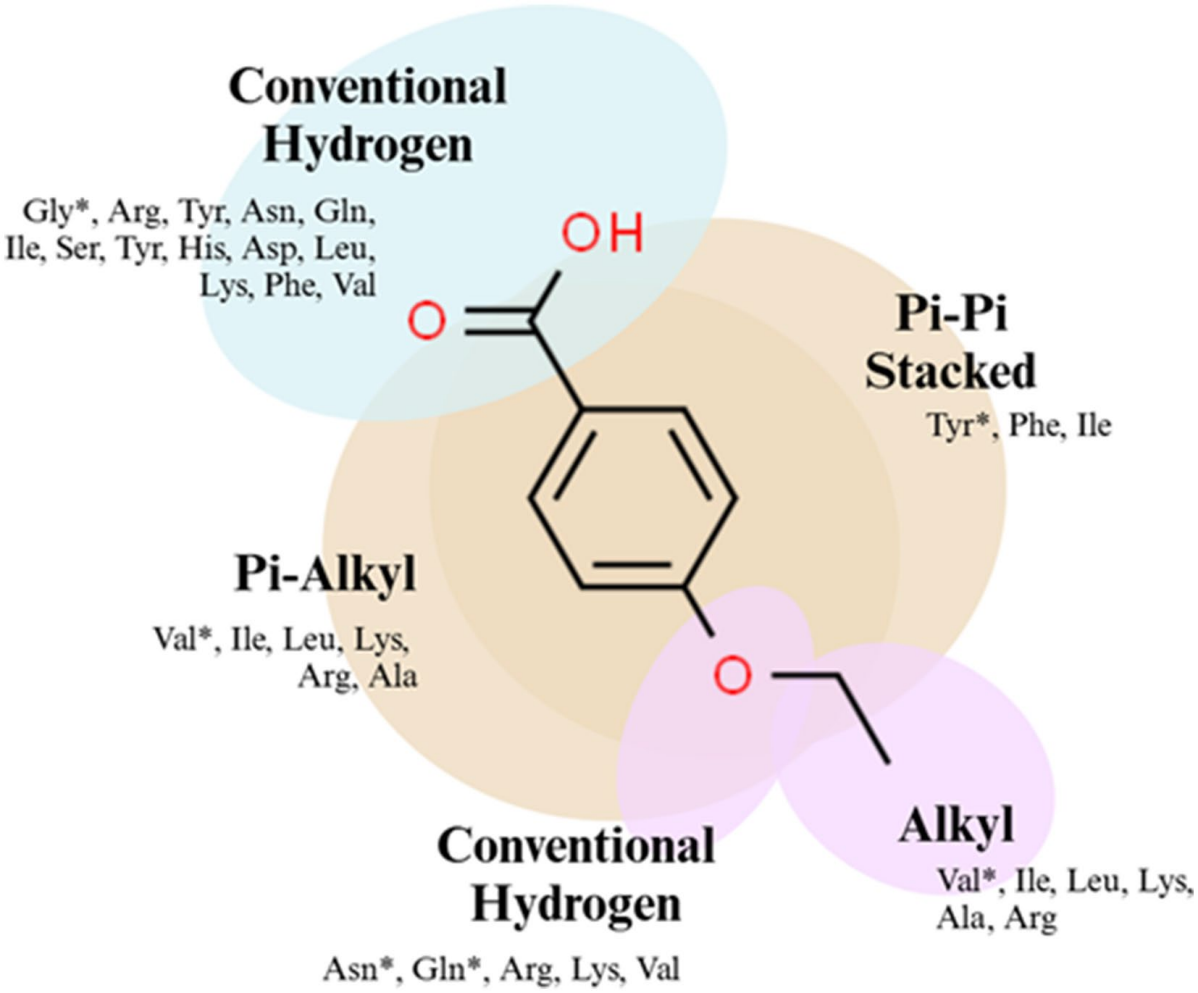
Graphical representation of 4EB binding affinities. *In silico* molecular docking analysis results revealed that 4EB binds favorably to hydrophobic residues within the proteins of interest. The amino acid residues that interacted with the 4EB molecule are listed, and residues that bind the most frequently are indicated by an asterisk (*).

**Figure 10:**
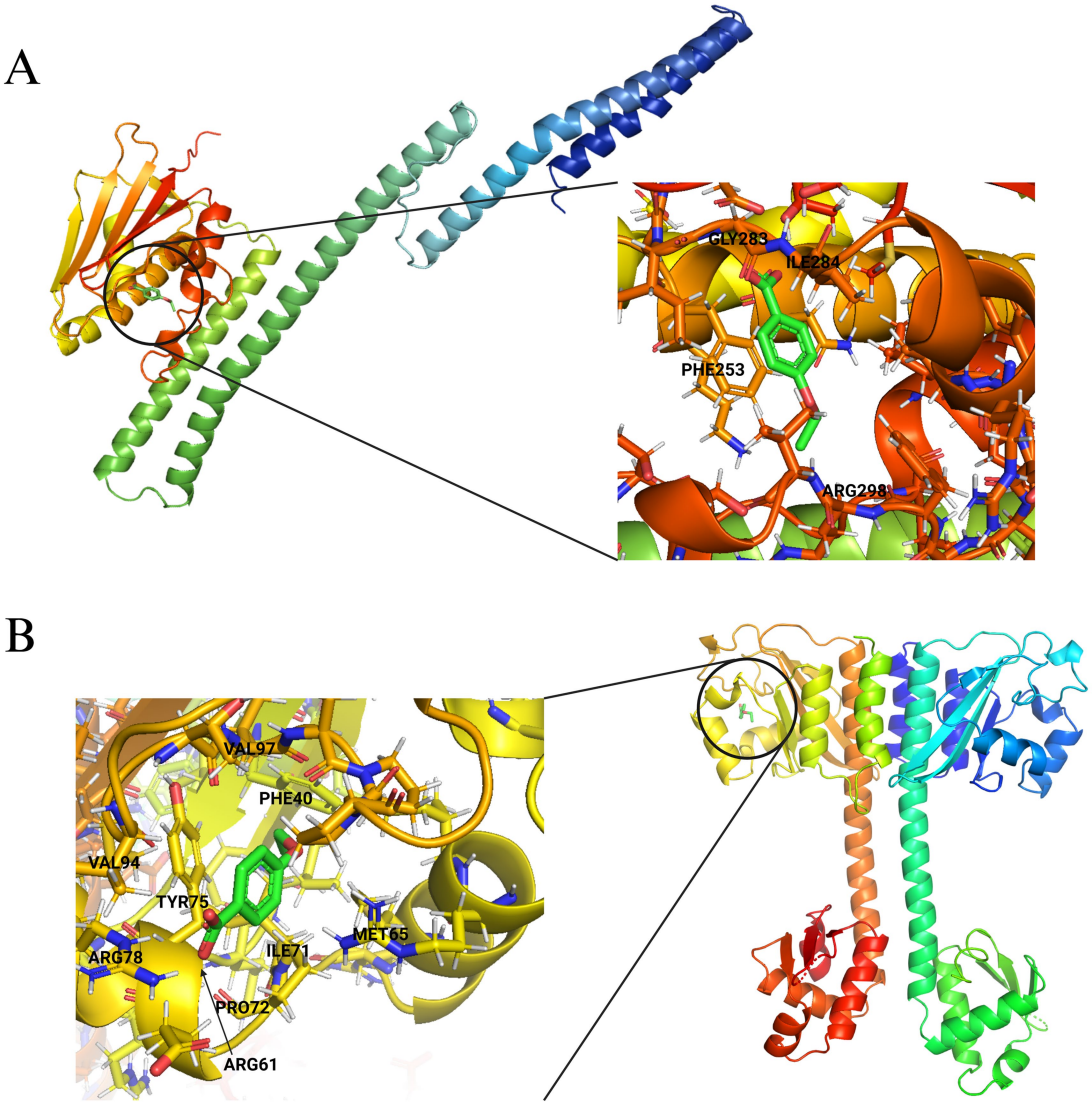
*In silico* molecular docking of **(A)** SaeS and **(B)** CodY with 4EB. 3D images are representations of the putative 4EB-binding pockets.

Based on the shared features among the 27 proteins with which 4EB associated most strongly, we hypothesized that these proteins potentially derived from a common ancestral structure or shared a conserved domain. To test this hypothesis, we aligned the 100 proteins of interest using a MUSCLE Multiple Sequence Alignment tool (Edgar, 2004) to visualize the protein phylogeny (Supplementary Figure 7). Some proteins, such as GraR, SarA, RsbU, and Nuc clustered within the phylogenetic tree. However, most of the proteins with the most favorable binding to 4EB were not clustered. The conserved domains for the 27 proteins were analyzed and were compared using the NCBI Conserved Domain Database (Yang et al., 2020). Neither common ancestry nor conserved domains were shared among all the 27 proteins with favorable ΔG values (Supplementary Figures 8A,B).

## 4 Discussion

Anti-virulence strategies are essential for controlling pathogens in an era of multidrug resistance. In previous work, we reported the anti-biofilm effects of several subinhibitory concentrations of 4EB, with 0.8 mg/mL 4EB reducing biofilm formation by 88% (Campbell et al., 2020). The present work confirmed that 0.8 mg/mL 4EB was the MIC_50_ (concentration attenuating 50% of planktonic growth relative to untreated controls) and that it inhibited no more than 18% of metabolic activity across all growth stages. The reason for the discrepancy in metabolic inhibition compared to growth inhibition is not currently understood, although 4EB may interfere with normal cell division processes including those dependent upon the WalK/WalR functions (cell wall metabolism (Dubrac et al., 2007)), as suggested by the *in silico* results showing favorable binding of 4EB within the histidine kinase conserved domain of WalK. Similarly, the quorum sensing inhibiting molecule Azan-7 in combination with clindamycin was found to significantly decrease biofilm formation by approximately 60% while minimally inhibiting overall metabolism (Bernabè et al., 2021), an observation that the authors suggested may be due to inhibition of the *icaABCDR* operon.

A differential effect of a high or a low concentration of 4EB on biofilm and virulence gene expression (Zhang et al., 2024) was evident at 3 h but not at 9 h. This pattern suggests that treatment with 0.8 mg/mL caused 4EB to penetrate the cell and reach its intracellular targets faster than treatment with 0.4 mg/mL, leading to a stronger impact on gene expression by 0.8 mg/mL 4EB. In contrast, by 9 h, the concentration-dependent difference was no longer evident, potentially because the intracellular targets of 4EB were fully saturated, regardless of the initial concentrations that were present. This finding may be relevant to preventing biofilm formation, where the early stages of surface attachment impact the progress to mature biofilms (Moormeier et al., 2014) and which in turn impact the success of *S. aureus* infections (Tran et al., 2023). These data are also consistent with earlier findings that treating with 0.8 mg/mL 4EB reduced biofilm formation by *S. aureus* to a greater extent than 0.4 mg/mL 4EB or lower concentrations (Campbell et al., 2020). Notably, in recent work on the impact of 4EB on *S. aureus* biofilm development, 0.8 mg/mL 4EB was found to significantly alter *S. aureus* biofilm structure and reduce overall biofilm formation at 6 h following inoculation, which corresponds to the multiplication phase of *S. aureus* biofilm development (Marchesani et al., 2025).

Due to the substantial prevention of biofilm formation and the moderate growth inhibition, 0.8 mg/mL 4EB was selected for further experimentation, with the intent of using the knowledge gleaned to identify more potent anti-virulence compounds in future work. *In vitro* experiments demonstrated for the first time that 4EB significantly downregulated diverse *S. aureus* virulence factors across several growth stages. *In silico* analysis predicted that 4EB binds to common features within several regulatory and non-regulatory proteins, notably interacting with valine and tyrosine residues in a putative 4EB binding pocket. The *in silico* findings were supported by *in vitro* gene expression measurements that validated the ability of 4EB to alter transcription of key virulence-related genes, indicating its potential as an anti-virulence agent.

4EB significantly reduced the production of Hla, an exoprotein that is essential for disease progression during host-pathogen interactions (Burlak et al., 2007), with a corresponding reduction in hemolysis. Transcriptional regulators AgrAC and SaeRS have been reported to impact Hla production (Schilcher et al., 2024; Liu et al., 2016; Gudeta et al., 2019; Pendleton et al., 2022). During 4EB treatment, the transcription of *agrA* was not significantly impacted at 15 h, but a significant downregulation of *saeR* was observed, consistent with the measured decrease in extracellular Hla. Hla has been shown to be essential for biofilm development in *S. aureus* (Caiazza and O'Toole, 2003). Additionally, molecular docking indicated that the sensor histidine kinase SaeS complexed effectively with 4EB within the conserved histidine kinase domain, suggesting that the observed downregulation of *saeS*, *saeR*, *sspA*, *hla* and *splC* and the significant reduction in exoprotein production all resulted from a reduction in SaeS activity. These findings are consistent with previous research that investigated the essential phosphorylation of SaeR by SaeS; if this phosphorylation step is inhibited, then the downstream transcription of *saeR*, *saeS*, *hla*, and exoprotein-related genes in the *sae*-regulon is nearly abolished (Mainiero et al., 2010; Liu et al., 2016).

The STXN carotenoid pigment has been extensively studied for its ability to provide resistance to reactive oxygen species (Vila et al., 2019; Liu et al., 2005; Antonic et al., 2013). The pigment is synthesized through a triterpenoid biosynthesis pathway that is encoded by the *crtMNOPQ* operon (Antonic et al., 2013). We observed that STXN production was decreased by 4EB treatment; however, we were initially perplexed by the finding that *crtM* was upregulated by 4EB. *In silico* analysis indicated that the decrease in STXN production was potentially due to 4EB complexing with and inhibiting the enzymatic function of the CrtN and CrtP proteins. Previous research has identified several STXN-inhibiting compounds, including compounds 5 m, a benzofuran analog (Wang et al., 2016), NP16, a sulfonamide derivative (Gao et al., 2017), and 1,4-benzodioxan derivatives (Ni et al., 2018) that are all predicted to inhibit CrtN activity. The interaction sites of these molecules with CrtN are currently unknown to the best of our knowledge. No CrtP inhibitors have been reported to date; the putative interaction between 4EB and CrtP warrants further investigation.

Three categories of pore-forming toxins are commonly present within *S. aureus*: alpha-toxin, leukocidins, and PSMs. We determined that 4EB treatment downregulated alpha-toxin and leukocidins and conversely upregulated α1-4*psm* and β1-2*psm* genes. During treatment with 4EB, *lukEvDv* transcription was downregulated approximately 500-fold during the log phase of growth. Elsewhere, transcription of the leukocidin genes *lukAB* was almost abolished in an *S. aureus* USA300 *saePQRS*-knockout mutant (Schilcher et al., 2024), indicating that the *sae*-operon is a direct transcriptional regulator of these virulence factors. Our results show that *saeR* and *saeS* transcription was substantially downregulated with 4EB, and *lukEv* and *lukDv* were both downregulated at levels in concordance with *hla*. These data suggest that *lukEvDv* are regulated by SaeRS in strain ATCC 6538. Enterotoxin (*entA* and *entD*) transcription patterns in response to 4EB were variable, consistent with other reports (Borst and Betley, 1994; Bayles and Iandolo, 1989). These findings highlight the complex regulatory network influenced by 4EB treatment.

The PSMs are small amphipathic proteins that have multiple biological activities, including host cell cytolysis and biofilm structuring (Periasamy et al., 2012; Wang et al., 2007). We determined that the α1-4 and β1-2*psm* genes were significantly upregulated during 4EB treatment, but *hld* (*δ*-toxin) transcription was not affected by 4EB. The *agr* operon has been identified as the transcriptional regulator of the *psm* genes and *hld* (Vuong et al., 2000; Cassat et al., 2006; Queck et al., 2008). Transcript levels for *hld* and *agrA* were not significantly affected during 4EB treatment; in contrast, the α1-4 and β1-2*psm* genes were significantly upregulated. These findings suggest that there is an additional transcriptional regulator, or possibly multiple transcriptional regulators, regulating α1-4 and β1-2*psm* genes that is independent of *agrA* transcriptional control. MgrA has been identified as a negative regulator of *psm* expression in strain NCTC8325 (Jiang et al., 2018); however, molecular docking indicated that 4EB does not form a favorable binding complex with MgrA. This suggests that MgrA inhibition may not be responsible for the upregulated expression of α1-4 and β1-2*psm* genes that were measured in this work. Previous studies have hypothesized that more transcriptional regulators of α*psm* and β*psm* operons may exist (Queck et al., 2008), although no additional regulators have been reported to date. Future work will investigate the divergent *psm* gene expression pattern observed in this study. Notably, this work identified the location of the α1-4 and β1-2*psm* coding regions within the ATCC 6538 genome (Supplementary Figure 8). Changes in PSM activity caused by 4EB may have contributed to the observed differences in biofilm structure reported here. The impact of the observed increase in *psm* transcription on *S. aureus* virulence was not evaluated in this work.

Molecular docking analysis identified nine transcriptional regulatory proteins that strongly complexed with 4EB (binding affinities of ΔG < −6.0 kcal/mol). Three of these were TCS proteins: WalK, SaeS, and GraS (Bleul et al., 2022). The *walKR* TCS is the only TCS that is essential for *S. aureus* growth (Villanueva et al., 2018; Monk et al., 2019), and it regulates cell-wall thickening in response to antibiotics as a form of antibiotic resistance (Howden et al., 2011). GraS is involved in cationic antimicrobial peptide (CAMP) resistance through the regulation of the virulence-associated *vraFG* operon (Dhankhar et al., 2020). GraR has been explored as a target of anti-virulence molecules (Dhankhar et al., 2020), but an inhibitory molecule targeting GraS has not yet been identified. Limited information exists regarding biofilm formation and the transcriptional regulators MalR, TreR, and YqfL, all of which formed favorable binding complexes with 4EB. Additionally, several non-regulatory virulence proteins were analyzed *in silico*. Notably, the Nuc (secreted nuclease, *nuc1*) protein has an important role in the *S. aureus* biofilm development process (Moormeier et al., 2014), and Nuc was determined to form a highly favorable binding complex with 4EB (ΔG = −6.28 kcal/mol). Of the nine regulatory proteins that were identified as 4EB targets *in silico*, *in vitro* measurements demonstrated that transcription of seven downstream regulons was significantly impacted by 4EB.

Molecular docking, phenotypic analysis, and *in vitro* validation suggested that for at least two proteins, 4EB acts as an agonist, increasing binding. Both IcaR and CodY transcriptional regulators formed favorable binding complexes with 4EB in silico, and the downstream regulons of IcaR (*icaA*) and CodY (*ilvD* and *oppB*) were significantly upregulated by 4EB. Two CodY promoter sites have been identified for *ilvD* and one CodY promoter site for *oppB* (Majerczyk et al., 2010). The intracellular adhesion *icaABCD* operon is required for biofilm formation in *S. aureus* (Cramton et al., 1999; Gerke et al., 1998; Heilmann et al., 1996), and the operon is regulated by the transcriptional repressor IcaR. When IcaR represses transcription of the *icaABCD* operon, the result is decreased biofilm formation (Yu et al., 2012; Yehia et al., 2022). 4EB inhibited biofilm formation (Campbell et al., 2020), and downregulated *icaA* transcript levels, suggesting that IcaR is activated by 4EB. CodY regulates the transcription of more than 200 genes, including many virulence factors (Majerczyk et al., 2008). Typically, *codY* knockout mutants have increased virulence factor production including biofilm formation and hemolysis (Majerczyk et al., 2008; Bulock et al., 2022). Molecular docking indicated that 4EB binds strongly with CodY, and in consideration of the measured phenotypes reported here in combination with the upregulation of *ilvD* and *oppB* expression *in vitro*, it is plausible that 4EB activates CodY.

Molecular docking analysis results revealed several notable features of 4EB’s interaction with *S. aureus* ATCC 6538 regulatory and virulence-associated proteins. First, there was a wide range of binding affinities, implying that 4EB associates with a subset of the evaluated proteins in a specific way. Second, there were common residues and protein structural features among the proteins that had the strongest association with 4EB. This result suggested that there is a putative 4EB binding site within these proteins. Among the proteins to which 4EB bound most strongly, it did not associate with residues that interact directly with DNA, indicating that the impact of 4EB is allosteric. Currently, the specific physical mechanism by which 4EB alters transcription is unknown. Third, the ability of 4EB to complex with a diverse set of transcriptional regulators is potentially a function of its small molecular size. This finding suggests a strategy by which a combination of two or more small molecules can be used simultaneously to inhibit *S. aureus* virulence. Notably, many plant-based essential oils with antimicrobial activity are combinations of multiple small molecules (Jafarı-sales and Pashazadeh, 2020; Gao et al., 2020; Han et al., 2021; Sreepian et al., 2022). Overall, a combination of *in vitro* and *in silico* analyses demonstrated that 4EB is a multi-mechanistic, anti-virulence and anti-biofilm compound. These findings can assist with understanding the types of molecular features that contribute to effective anti-virulence activity.

## Data Availability

The raw data supporting the conclusions of this article will be made available by the authors, without undue reservation.

## Footnotes

1
https://atcc.org

2
https://bioinformatics.org
