# Supplementary material for: The anti-biofilm compound 4-ethoxybenzoic acid inhibits Staphylococcus aureus virulence factor production via a putative 4EB-binding pocket in key virulence-associated proteins
Source: Front Microbiol. 2026 Feb 2;16:1704290. doi: 10.3389/fmicb.2025.1704290 (PMC12907329; doi:10.3389/fmicb.2025.1704290)
Supplement: Supplementary file 1 [file Data_Sheet_1.docx]

Supplementary Material

# Supplementary Methods

## Biofilm Flow Cell Construction and Imaging

Flow cells for non-destructive imaging of biofilms were constructed using 0.8 mm internal diameter peroxide-cured silicone tubing (Pharmed), Luer chromatography fittings (Bio-Rad), Masterflex peristaltic pumps (Cole Parmer) and 3-stop tubing (Pharmed) (1). Commercially available silicone adhesive was used to attach 22 x 60 cm glass microscope cover slips (Fisherbrand) to either end of a 76 x 25 x 2 cm piece of Delrin plastic sheeting with two channels cut into it to form an airtight seal and with 1.0 mm stainless steel tubing inserted through holes drilled at the ends to accommodate tubing for influent and effluent media. LB broth or LB broth supplemented with 0.8 mg/mL 4EB were used.

Flow cells were sterilized with 10% bleach for 10 minutes, followed with 10 minutes of pumping autoclaved DDI water to rinse. All experiments began with a normalized OD_600_ value of 0.03, diluted from an overnight culture. To seed the flow cells with bacteria, cells were injected 10-20 cm upstream of the flow cell channel as previously described using a 1 mL Luer lock syringe (Fisherbrand) and 22G (0.7 mm x 25 mm) BD PrecisionGlide Needles (2). After injection, pumps were started for 20-30 seconds to prevent upstream contamination. Then, pumps were stopped for 1 hour to allow for adhesion before growing for 24 hours with a flowrate of 0.3 mL/min. The temperature (37 °C) was maintained by placing the flow cells on a support inside a water bath.

Prior to imaging, 1X PBS was pumped into the flow cell at 0.3 mL/min for 15 minutes. Next, 1 mL of acridine orange (100 ppm) was injected 10-20 cm upstream of the flow cell chamber, following the same protocol described above, and allowed to sit for 10 minutes. Biofilms were rinsed with 1x PBS again for 15 minutes before imaging to remove excess dye. An LSM510 confocal laser scanning microscope (Zeiss) was used to capture all images at 100× magnification. Zeiss Zen Lite software was used to visualize and quantify the biofilms.

# Supplementary Figures

**
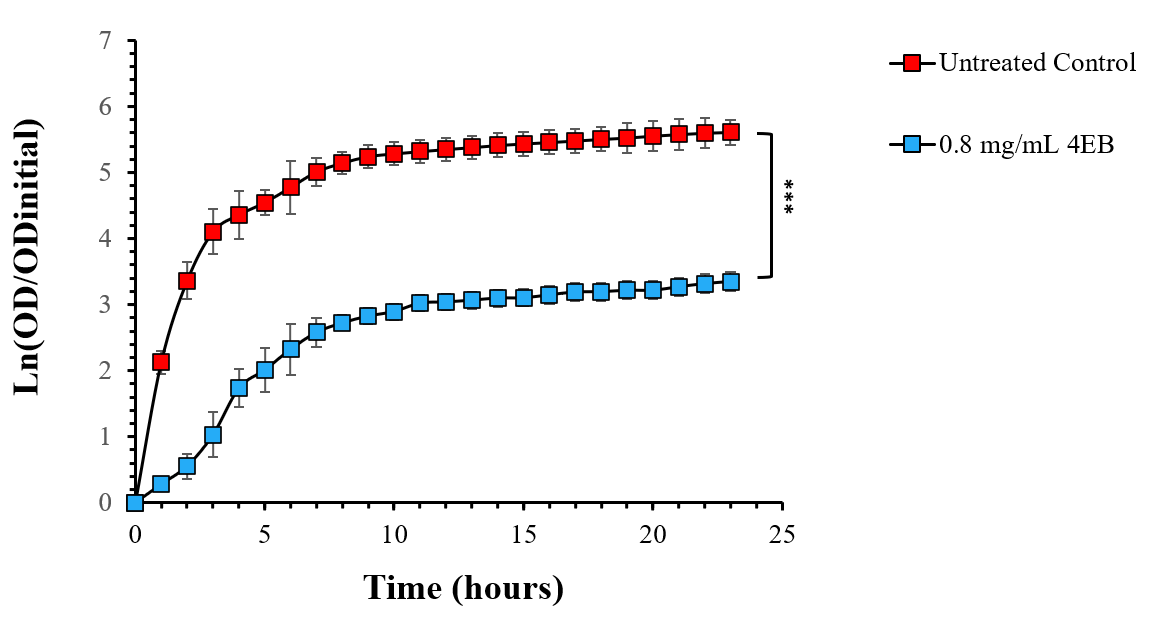
**

**Supplementary Figure 1: Growth of *S. aureus* ATCC 6538 during treatment with 0.8 mg/mL 4EB.** Growth of *S. aureus* was evaluated for 24 hours during treatment with 4EB and the untreated control. Statistical significance: ***, *P* < 0.001.


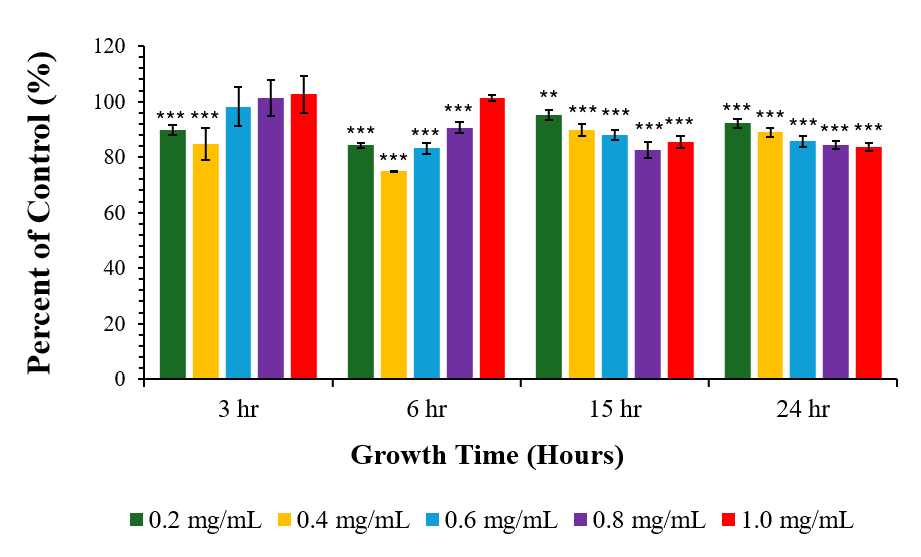


**Supplementary Figure 2: Metabolic activity of *S. aureus* during treatment with 4EB across several growth stages.** The resazurin assay was used as an indicator of cell metabolic activity. Resorufin fluorescence was measured over time for *S. aureus* cells treated with several concentrations of 4EB. The fluorescence for each 4EB treatment per timepoint was used to calculate the average percentage of metabolic activity compared to the untreated control. Statistical significance: *, *P* < 0.05; **, *P* < 0.01; ***, *P* < 0.001.


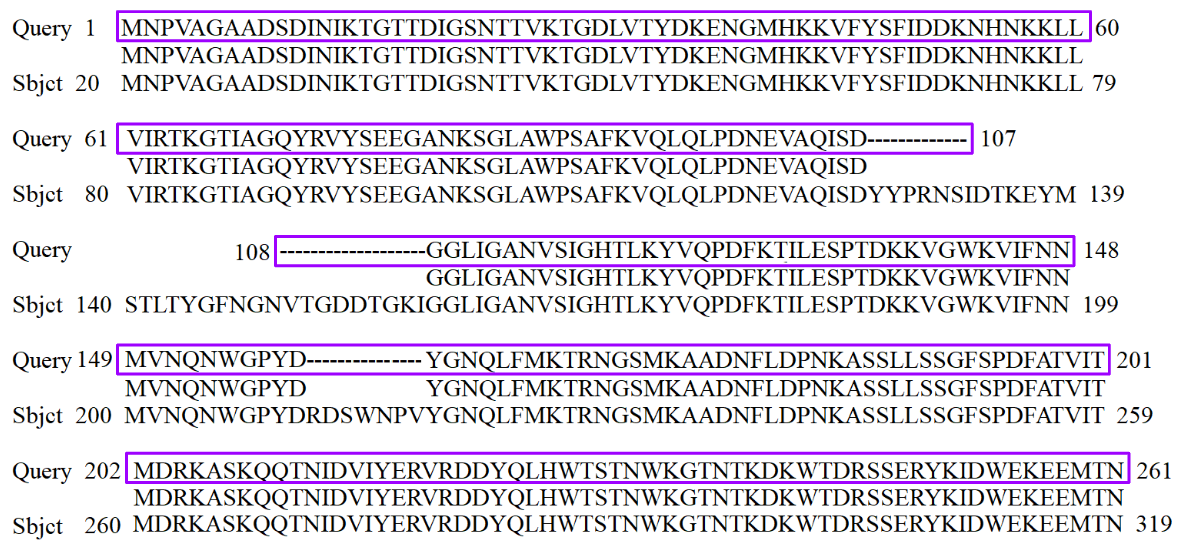


**Supplementary Figure 3: Hla amino acid sequence.** The peptide sequence was aligned using the NCBI BLASTP database, and the sequence was identified as the *S. aureus sp.* alpha-hemolysin protein (86.7% identity match).


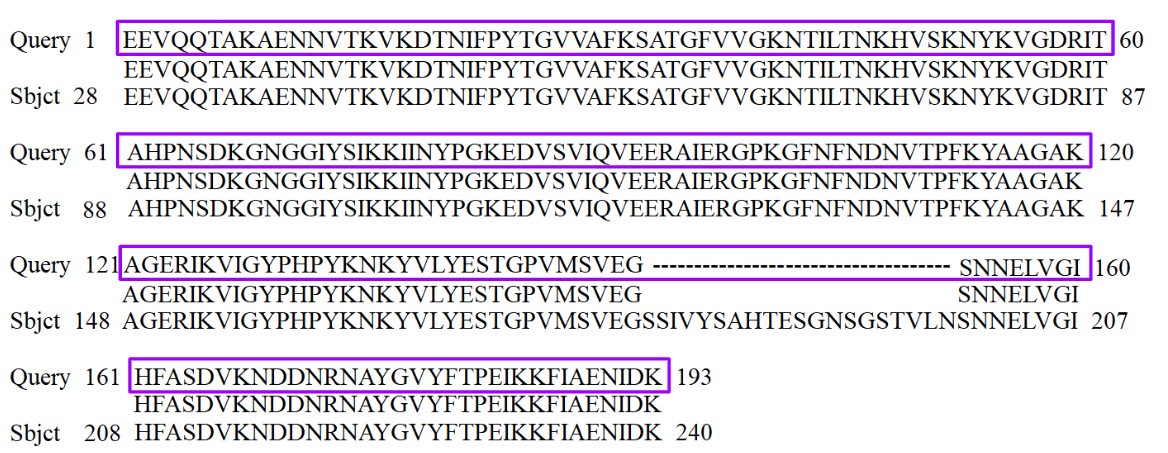


**Supplementary Figure 4: SplC amino acid sequence.** The peptide sequence was aligned using the NCBI BLASTP database, and the sequence was identified as the *S. aureus sp.* serine-like protease C protein (90.6% identity match).

**A**


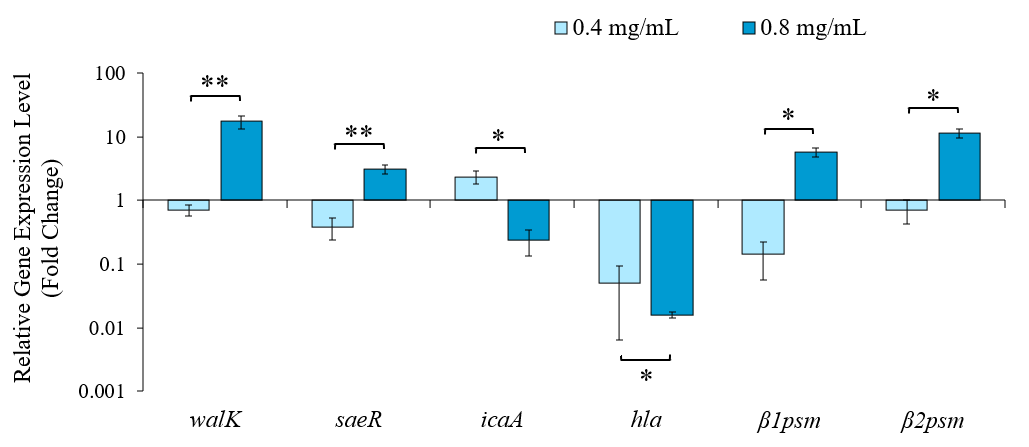


**B**


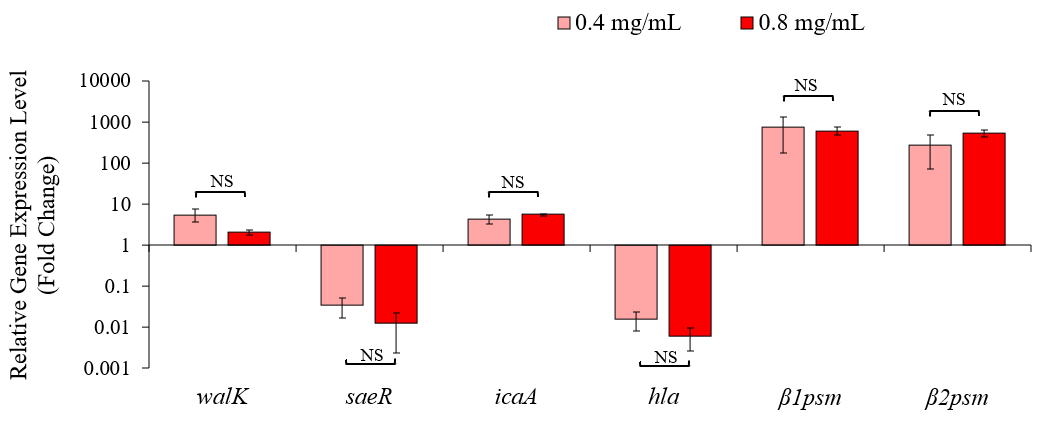


**Supplementary Figure 5: RT-qPCR analysis of biofilm- and virulence-associated genes in response to concentrations of 4EB.** A) Expression of genes at 3 hours of growth during treatment with 0.4 and 0.8 mg/mL 4EB. B) Expression of genes at 9 hours of growth during treatment with 0.4 and 0.8 mg/mL 4EB. The results represent the mean ± SEM of the triplicate RT-qPCR determinations of each cDNA sample obtained from three replicate planktonic cultures. Statistical significance: *, P < 0.05; **, P < 0.01; ***, P < 0.001.


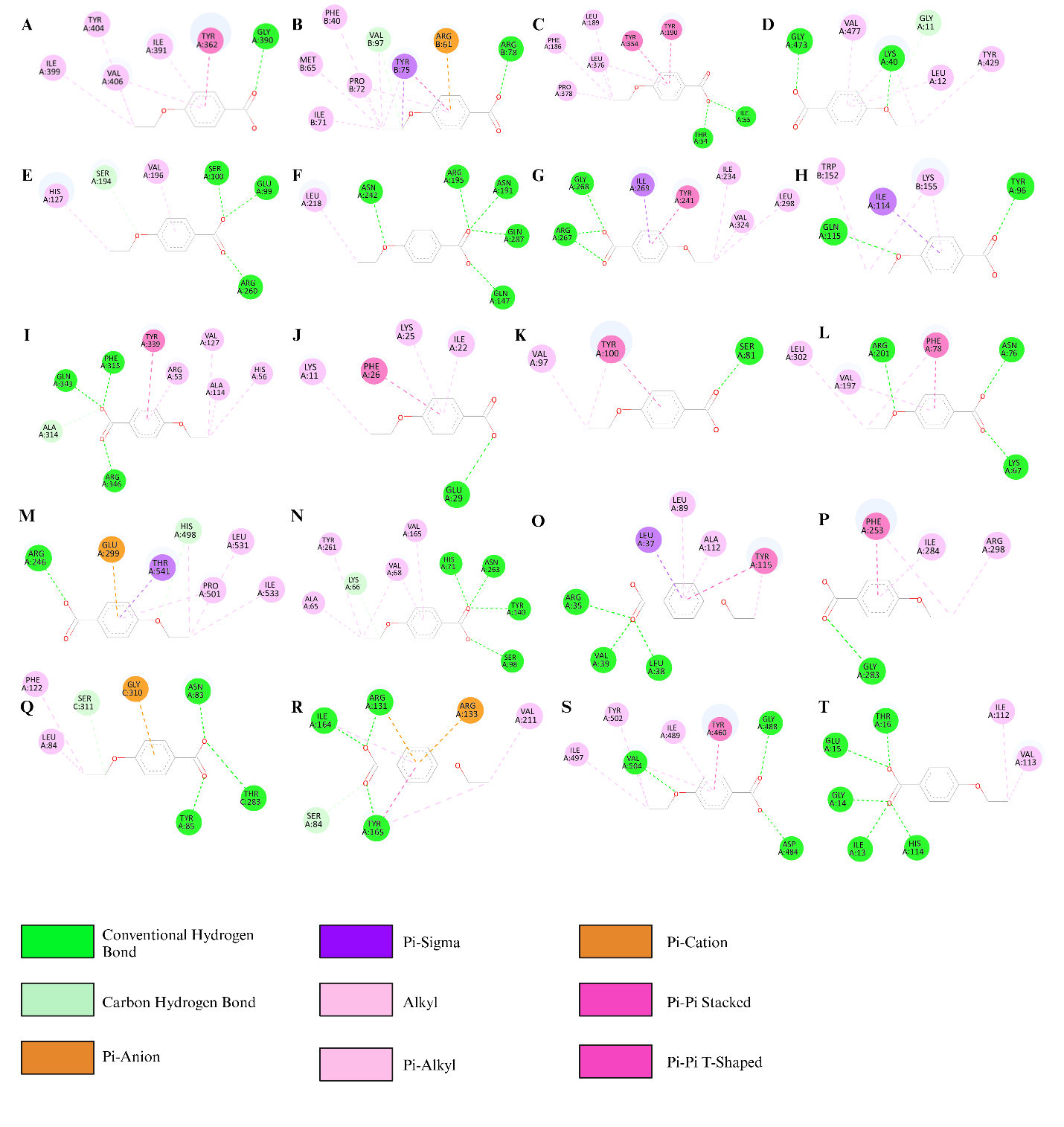


**Supplementary Figure 6: Optimal binding complexes showing the most favorable conformations with 4EB.** Proteins: A) ArlS, B) CodY, C) CrtN, D) CrtP, E) CynR, F) DegA, G) GraS, H) IcaR, I) KatA, J) KPFDPMNM_01268, K) KPFDPMNM_01724, L) MalR, M) ManR, N) MsrR, O) Nuc, P) SaeS, Q) SdrC, R) TreR, S) WalK, T) YqfL.


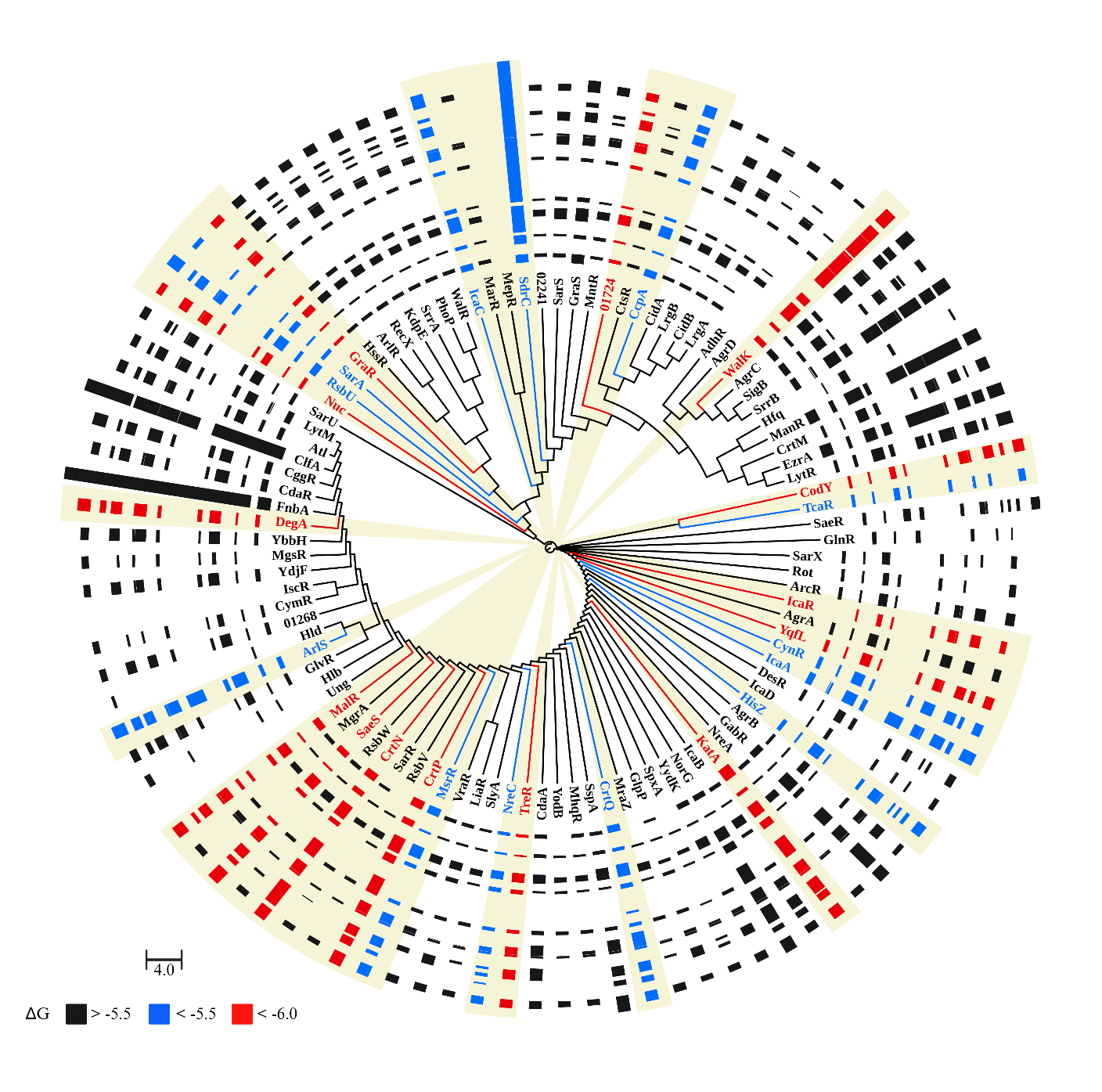


**Supplementary Figure 7: Phylogenetic tree relating 100 virulence-associated proteins within *S. aureus* ATCC 6538 that were analyzed *in silico* by molecular docking with 4EB.** The red (ΔG < -6.0 kcal/mol) and blue (ΔG < -5.5 kcal/mol) highlighting represents favorable binding. Binding affinities were estimated using Autodock Vina and the phylogenetic tree was constructed with MUSCLE; see Methods for details. CLUSTAL multiple sequence protein alignments are shown.


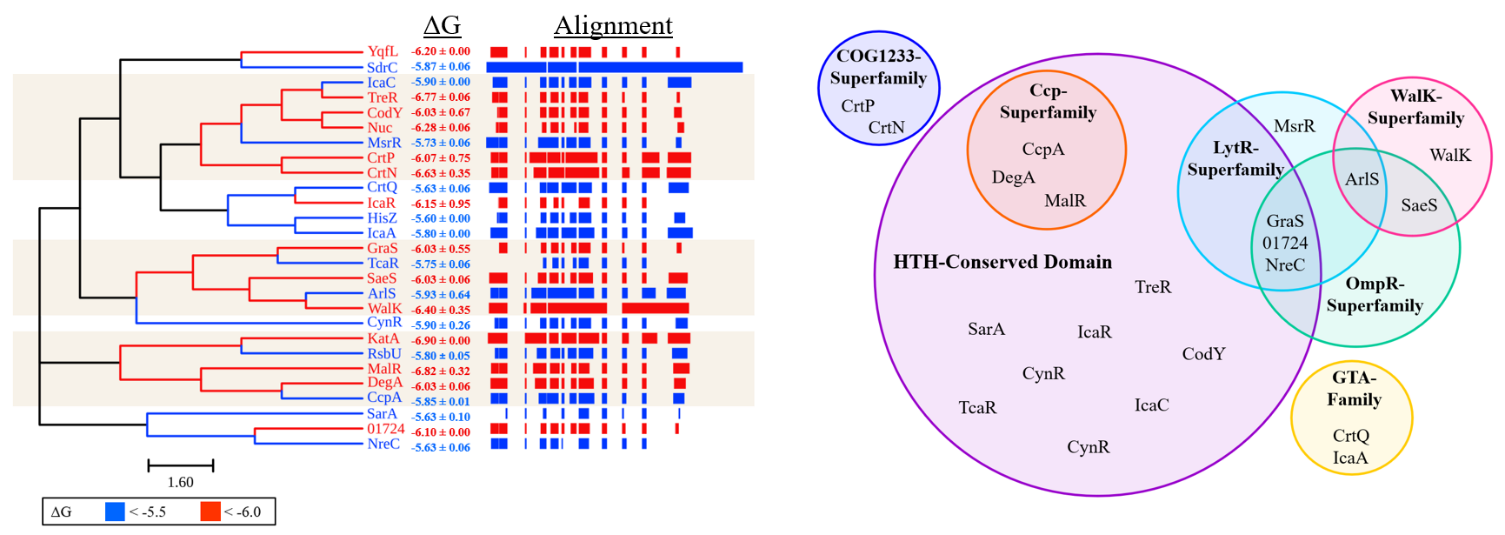


A

B

**Supplementary Figure 8: Proteins that formed highly favorable complexes (ΔG < -6.0 kcal/mol) with 4EB from *in silico* experiments.** A) Phylogenetic tree, ΔG binding affinities with 4EB (kcal/mol), and peptide sequence alignments. B) Conserved domains and superfamilies of the proteins. Proteins not listed within the circles were found to not have any common conserved domains or superfamilies in common with the proteins listed here.

| **Gene** | **Nucleotide sequence (5’ 🡪 3’)** | **bp** |
| --- | --- | --- |
| α1 *psm* | ATGGGTATCATCGCTGGCATCATTAAAGTTATCAAAAGCTTAATCGAACA  ATTCACTGGTAAATAA | 66 |
| α2 *psm* | ATGGGTATCATTGCAGGAATCATTAAATTCATTAAAGGATTAATTGA  GAAATTCACTGGTAAG | 63 |
| α3 *psm* | ATGGAATTCGTAGCAAAATTATTCAAATTCTTTAAAGATTTACTTGG  TAAATTTTTAGGTAACAAC | 66 |
| α4 *psm* | ATGGCTATTGTAGGTACTATCATTAAAATCATCAAAGCAATTATCGA  CATTTTCGCAAAATAATTTAAG | 69 |
| β1 *psm* | ATGACTGGACTAGCAGAAGCAATCGCAAATACTGTGCAAGCTGCA  CAACAACATGATAGTGTGAAATTAGGCACAAGTATCGTAGACATCG  TTGCTAACGGTGTGGGTTTACTAGGTAAATTATTTGGATTCTAA | 129 |
| β2 *psm* | ATGGAAGGTTTATTTAACGCAATTAAAGATACCGTAACTGCAGCAA  TTAATAATGATGGCGCAAAATTAGGCACAAGCATTGTGAGCATCGT  TGAAAATGGCGTAGGTTTATTAGGTAAATTATTCGGATTCTAA | 135 |
| δ *psm* | ATGAGTTGTTTAATTTTAAGAATTTTTATCTTAATTAAGGAAGGAGTG  ATTTCAATGGCACAAGATATCATTTCAACA | 78 |

**Supplementary Table 1: Nucleotide sequences of *psm* genes encoded within the *S. aureus* ATCC 6538 genome.**

| ***Protein Name***  A | ***Average ΔG Binding Affinity***  ***(kcal/mol)*** | ***Protein Function*** |
| --- | --- | --- |
| MalR | -6.82 ± 0.32 | HTH-type transcriptional regulator; maltose metabolism |
| TreR | -6.77 ± 0.06 | HTH-type transcriptional regulator; trehalose metabolism |
| WalK | -6.40 ± 0.95 | Sensor protein kinase |
| IcaR | -6.15 ± 0.35 | HTH-type negative transcriptional regulator; biofilm operon *icaADBC* |
| YqfL | -6.20 ± 0.00 | Putative pyruvate, phosphate dikinase regulator |
| KPFDPMNM_01724 | -6.10 ± 0.00 | Putative response regulatory protein |
| CodY | -6.03 ± 0.67 | GTP-sensing transcriptional pleiotropic repressor |
| DegA | -6.03 ± 0.06 | HTH-type transcriptional regulator |
| GraS | -6.03 ± 0.55 | Sensor histidine kinase |
| SaeS | -6.03 ± 0.67 | Histidine protein kinase |
| ArlS | -5.93 ± 0.64 | Signal transduction histidine-protein kinase |
| CcpA | -5.85 ± 0.01 | Catabolite control protein |
| CynR | -5.90 ± 0.26 | HTH-type transcriptional regulator |
| SdrC | -5.87 ± 0.06 | Serine-aspartate repeat-containing protein C |
| TcaR | -5.75 ± 0.06 | HTH-type regulator |
| MsrR | -5.73 ± 0.06 | Regulatory protein; cell wall associated |
| RsbU | -5.70 ± 0.17 | Phosphoserine phosphatase |
| NreC | -5.63 ± 0.06 | Oxygen regulatory protein |
| SarA | -5.63 ± 0.10 | Regulatory protein; virulence genes regulator |
| HisZ | -5.60 ± 0.00 | ATP phosphoribosyltransferase regulatory subunit |
| PhoP | -5.53 ± 0.12 | Alkaline phosphatase synthesis transcriptional regulator |
| GabR | -5.50 ± 0.21 | HTH-type transcriptional regulator |
| ManR | -5.50 ± 0.00 | Regulatory protein |
| SarS | -5.50 ± 0.15 | HTH-type transcriptional regulator |
| AgrB | -5.43 ± 0.06 | Accessory gene regulator protein B |
| CggR | -5.43 ± 0.06 | Central glycolytic gene regulator |
| KPFDPMNM_01268 | -5.43 ± 0.75 | Putative transcription regulator |
| SarU | -5.37 ± 0.06 | HTH-type transcriptional regulator |
| Rot | -5.35 ± 0.06 | HTH-type transcriptional regulator |
| DesR | -5.33 ± 0.38 | Transcriptional regulatory protein |
| WalR | -5.33 ± 0.06 | Transcriptional regulatory protein |
| MntR | -5.30 ± 0.00 | Transcriptional regulatory protein |
| YbbH | -5.30 ± 0.26 | Putative HTH-type regulatory protein |
| NorG | -5.20 ± 0.00 | HTH-type transcriptional regulator |
| AdhR | -5.17 ± 0.06 | HTH-type transcriptional regulator |
| YydK | -5.17 ± 0.06 | Putative HTH-type transcriptional regulator |
| CtsR | -5.10 ± 0.00 | Transcriptional regulator |
| GraR | -5.10 ± 0.00 | Response regulatory protein |
| SrrA | -5.13 ± 0.21 | Transcriptional regulatory protein |
| VraR | -5.10 ± 0.12 | Response regulatory protein |
| YodB | -5.10 ± 0.00 | HTH-type transcriptional regulator |
| MgrA | -5.08 ± 0.06 | HTH-type transcriptional regulator |
| EzrA | -5.00 ± 0.00 | Septation ring formation regulator |
| MraZ | -4.97 ± 0.06 | Transcriptional regulator |
| SlyA | -4.97 ± 0.06 | Transcriptional regulator |
| HssR | -4.93 ± 0.06 | Heme response regulator |
| SigB | -4.93 ± 0.06 | RNA polymerase sigma-B factor |
| LytR | -4.88 ± 0.15 | Transcriptional regulator |
| SarX | -4.85 ± 0.06 | HTH-type transcriptional regulator |
| SarR | -4.83 ± 0.06 | HTH-type transcriptional regulator |
| ArcR | -4.80 ± 0.00 | HTH-type transcriptional regulator |
| IscR | -4.80 ± 0.00 | Iron-sulfur cluster regulator |
| GlnR | -4.73 ± 0.12 | HTH-type transcriptional regulator |
| GlvR | -4.73 ± 0.15 | HTH-type transcriptional regulator |
| MhqR | -4.73 ± 0.06 | HTH-type transcriptional regulator |
| SaeR | -4.73 ± 0.12 | Response regulator |
| YdjF | -4.73 ± 0.06 | Putative HTH-type transcriptional regulator |
| GlpP | -4.70 ± 0.00 | Glycerol uptake operon anti-terminator regulatory protein |
| LiaR | -4.67 ± 0.06 | Transcriptional regulatory protein |
| KPFDPMNM_02241 | -4.63 ± 0.12 | Putative HTH-type transcriptional regulator |
| CymR | -4.60 ± 0.00 | HTH-type transcriptional regulator |
| KdpE | -4.53 ± 0.06 | *kdp*-operon transcriptional regulatory protein |
| RecX | -4.53 ± 0.06 | Regulatory protein |
| AgrA | -4.47 ± 0.21 | Accessory gene regulator A |
| AgrC | -4.47 ± 0.12 | Sensor protein kinase |
| SpxA | -4.47 ± 0.06 | Regulatory protein |
| MgsR | -4.30 ± 0.10 | Regulatory protein |
| ArlR | -4.23 ± 0.00 | Response regulator |
| CdaR | -4.10 ± 0.10 | *cdaA* regulatory protein |
| AgrD | -3.97 ± 0.06 | Accessory gene regulator D |
| MarR | -2.23 ± 0.06 | Transcriptional repressor |
| MepR | -2.18 ± 0.06 | Multidrug export regulatory protein |

B

| ***Protein Name*** | ***Average ΔG Binding Affinity***  ***(kcal/mol)*** | ***Protein Function*** |
| --- | --- | --- |
| KatA | -6.90 ± 0.00 | Catalase |
| CrtN | -6.63 ± 0.35 | Dehydrosqualene desaturase; Staphyloxanthin synthesis |
| Nuc | -6.28 ± 0.06 | Nuclease |
| CrtP | -6.07 ± 0.75 | Diapolycopene oxygenase; Staphyloxanthin synthesis |
| IcaC | -5.90 ± 0.00 | putative poly-beta-1,6-N-acetyl-D-glucosamine export protein |
| IcaA | -5.80 ± 0.00 | Poly-beta-1,6-N-acetyl-D-glucosamine synthase |
| CrtQ | -5.63 ± 0.06 | 4,4'-diaponeurosporenoate glycosyltransferase; Staphyloxanthin synthesis |
| NreA | -5.53 ± 0.06 | Hypothetical protein |
| Atl | -5.40 ± 0.00 | Bifunctional autolysin |
| ClfA | -5.33 ± 0.12 | Clumping factor A |
| LytM | -5.20 ± 0.00 | Glycyl-glycine endopeptidase |
| IcaB | -5.17 ± 0.29 | Poly-beta-1,6-N-acetyl-D-glucosamine N-deacetylase |
| SdrE | -5.17 ± 0.06 | Serine-aspartate repeat-containing protein E |
| CdaA | -5.10 ± 0.00 | Cyclic di-AMP synthase |
| FnbA | -5.00 ± 0.00 | Fibronectin-binding protein A |
| LrgB | -4.83 ± 0.06 | Antiholin-like protein |
| RsbW | -4.83 ± 0.06 | Serine-protein kinase |
| IcaD | -4.80 ± 0.00 | Poly-beta-1,6-N-acetyl-D-glucosamine synthesis protein |
| CidB | -4.60 ± 0.10 | Holin-like protein B |
| Hlb | -4.60 ± 0.00 | Phospholipase C; Beta-hemolysin |
| CidA | -4.57 ± 0.06 | Holin-like protein A |
| SspA | -4.57 ± 0.15 | Glutamyl endopeptidase |
| Hfq | -4.40 ±0.00 | RNA-binding protein |
| LrgA | -4.33 ± 0.06 | Antiholin-like protein |
| RsbV | -3.87 ± 0.06 | Anti-sigma-B factor antagonist |
| Hld | -3.80 ± 0.01 | Delta-hemolysin |

**Supplementary Table 2: *S. aureus* ATCC 6538 proteins of interest for *in silico* experiments.** The tables show the ΔG ± standard deviation results for A) transcriptional regulatory proteins, and B) other virulence-related enzymes.

| **Protein Name** | **Protein Function** | **Average ΔG (kcal/mol)** | **Regulated Genes** | **Gene Function** | **Relative Expression (Fold Change)** |
| --- | --- | --- | --- | --- | --- |
| MalR | HTH-type transcriptional regulator; maltose metabolism | -6.82 ± 0.32 | *malR* | Transcriptional regulator; maltose metabolism (3) | -5.6 |
|  |  |  | *malL* | Oligo-1,6-glucosidase (4) | -5.5 |
| TreR | HTH-type transcriptional regulator; trehalose metabolism | -6.77 ± 0.06 | *treR* | Transcriptional regulator; trehalose metabolism (5) | -4.2 |
|  |  |  | *treA* | Trehalose-6-phosphate hydrolase (5) | -5.1 |
| WalK | Sensor protein kinase; cell wall regulation | -6.40 ± 0.95 | *walK* | Sensor histidine kinase; cell wall regulation (6) | -1.3 |
|  |  |  | *walR* | Transcriptional regulatory protein; cell wall regulation (6) | -1.4 |
|  |  |  | *lytM* | Glycyl-glycine endopeptidase (6) | -2.8 |
| IcaR | HTH-type negative transcriptional regulator; biofilm operon | -6.15 ± 0.35 | *icaA* | Poly-beta-1,6-N-acetyl-D-glucosamine synthase (7) | -8.5 |
| YqfL | Putative pyruvate phosphate dikinase regulator | -6.20 ± 0.00 | *yqfL* | Putative pyruvate phosphate dikinase regulator (8) | -1.5 |
|  |  |  | *ccpN-1* | Transcriptional repressor; gluconeogenesis (8) | -1.6 |
|  |  |  | *ccpN-2* |  | -1.8 |
| CodY | GTP-sensing transcriptional pleiotropic repressor | -6.03 ± 0.67 | *ilvD* | Dihydroxy-acid dehydratase (9) | 2.2 |
|  |  |  | *oppB* | Oligopeptide transport system permease protein (10) | 3.3 |
| DegA | HTH-type transcriptional regulator; inositol metabolism | -6.03 ± 0.06 | *degA* | HTH-type transcriptional regulator; inositol metabolism (11) | -2.2 |
|  |  |  | *iolG* | Inositol 2-dehydrogenase/D-chiro-inositol 3-dehydrogenase (11) | -3.5 |
| GraS | Sensor histidine kinase; host defense peptide resistance | -6.03 ± 0.55 | *graS* | Sensor histidine kinase; host defense peptide resistance (12) | -17.7 |
|  |  |  | *graR* | Response regulatory protein; host defense peptide resistance (12) | -87.2 |
| SaeS | Histidine protein kinase; exoprotein production | -6.03 ± 0.67 | *saeS* | Sensor histidine kinase; exoprotein production (13) | -11.2 |
|  |  |  | *saeR* | Response regulatory protein; exoprotein production (13) | -33.4 |
|  |  |  | *emp* | Extracellular matrix protein-binding protein (14) | -12.6 |

**Supplementary Table 3: Protein targets of 4EB and the downstream genes that they regulate.** The table includes the nine proteins that were predicted to be affected by 4EB using *in silico* analysis (Column: Average ΔG (kcal/mol)) alongside the protein functions. The downstream gene regulons of the proteins are listed alongside the gene functions. The *in vitro* analysis results are the average of the triplicate RT-qPCR determinations of each cDNA sample obtained from three replicate planktonic cultures at 9 hr.

|  | **Known Binding Sites (Amino Acid residue #)** | | | | |  |
| --- | --- | --- | --- | --- | --- | --- |
|  | **DNA-Binding** | **Enzymes** | | **Sensor Kinase Proteins** | |  |
| **Protein** | **HTH-Motif** | **Ligand Binding Sites** | **Active Sites** | **Transmembrane Domains** | **Histidine Kinase** | **Sources** |
| MalR | 2 – 56 |  |  |  |  | UniProt: A0A0H3JSG1 |
| TreR | 31 – 50 |  |  |  |  | UniProt: P39796 |
| WalK |  | Zn^2+^: 271, 274, 364, 368 |  | 14 – 34  183 – 203 | 382 – 600 | UniProt: A6QD58 (15) |
| IcaR | 21 – 41 |  |  |  |  | UniProt: Q5HCN2 |
| YqfL | Putative, no information currently available | | | | | |
| KPFDPMNM_01724 | Putative, no information currently available | | | | | |
| CodY | 203 – 222 |  |  |  |  | UniProt: Q2FHI3 (16) |
| DegA | 4 – 59 |  |  |  |  | UniProt: A0A0D3Q452 |
| GraS |  |  |  | 17 – 36, 42 – 63 | 126 – 332 | UniProt: A0A2K4AKQ7 |
| SaeS |  |  |  | 9 – 29, 40 – 60 | 129 – 348 | UniProt: Q2YSM6 |
| KatA |  | Fe: 339 | 56, 129 |  |  | UniProt: Q9L4S1 |
| CrtN | No information currently available | | | | | |
| Nuc |  | Ca^+^: 103, 122, 123 | 117, 125, 169 |  |  | UniProt: P00644 (17) |
| CrtP | No information currently available | | | | | |

**Supplementary Table 4: Binding site characteristics and active sites within proteins that formed highly favorable binding complexes with 4EB.** The numbers represent the amino acid residue location within each protein sequence. Proteins MalR, TreR, IcaR, CodY, and DegA are DNA-binding transcriptional regulators with the helix-turn-helix (HTH) DNA-binding motif. Proteins WalK, GraS, and SeaS are sensor kinase proteins (part of TCS systems) that regulate transcription through the phosphorylation of DNA-binding proteins. Proteins KatA, Nuc, CrtN, and CrtP are virulence-associated enzymes.


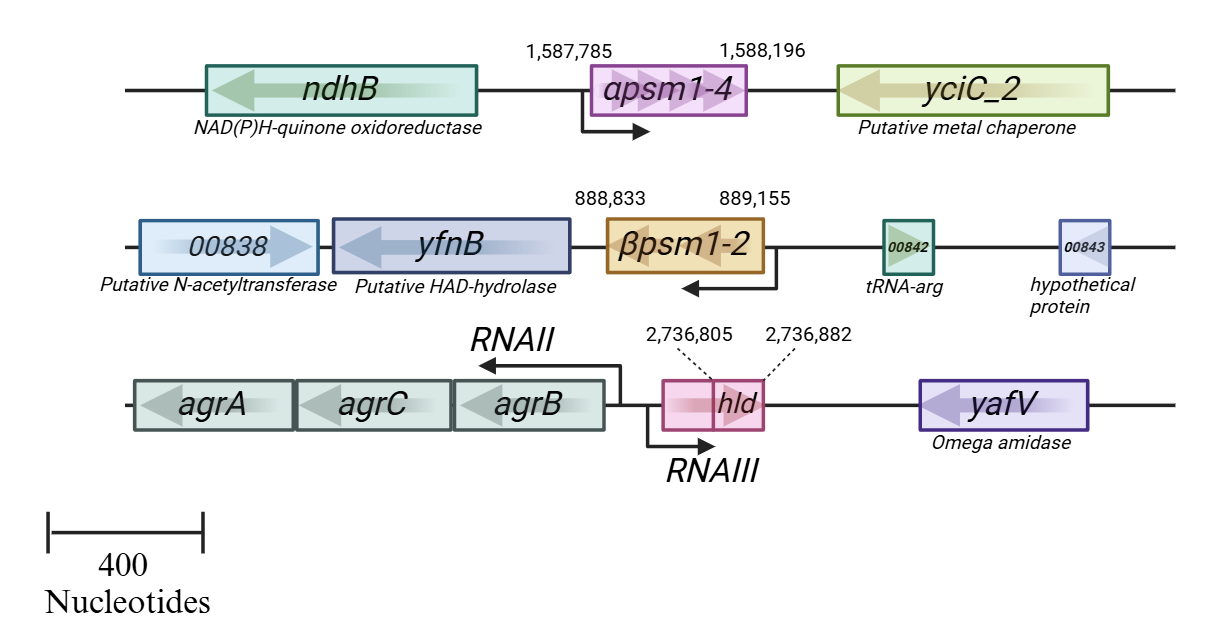


**Supplementary Figure 8: Phenol-soluble modulin (PSM) genetic coding regions located within the *S. aureus* ATCC 6538 genome.** Other genes located upstream and downstream the *psm* genes are shown. All genes and DNA coding regions are depicted to-scale (bottom left corner).

# Supplementary References

1. Gilbert ES, Keasling JD. Bench scale flow cell for nondestructive imaging of biofilms. InEnvironmental microbiology: methods and protocols 2004 Jul 15 (pp. 109-118). Totowa, NJ: Humana Press.

2. Crusz SA, Popat R, Rybtke MT, Camara M, Givskov M, Tolker-Nielsen T, et al. Bursting the bubble on bacterial biofilms: a flow cell methodology. Biofouling. 2012;28(8):835-42.

3. Andersson U, Rådström P. Physiological function of the maltose operon regulator, MalR, in Lactococcus lactis. BMC Microbiology. 2002;2(1):28.

4. Delgado S, Flórez AB, Guadamuro L, Mayo B. Genetic and biochemical characterization of an oligo-α-1, 6-glucosidase from Lactobacillus plantarum. International Journal of Food Microbiology. 2017;246:32-9.

5. Baker J, Lindsay E, Faustoferri R, To T, Hendrickson E, He X, et al. Characterization of the trehalose utilization operon in Streptococcus mutans reveals that the TreR transcriptional regulator is involved in stress response pathways and toxin production. Journal of Bacteriology. 2018;200(12):10.1128/jb. 00057-18.

6. Dubrac S, Boneca IG, Poupel O, Msadek T. New insights into the WalK/WalR (YycG/YycF) essential signal transduction pathway reveal a major role in controlling cell wall metabolism and biofilm formation in Staphylococcus aureus. Journal of Bacteriology. 2007;189(22):8257-69.

7. Cue D, Lei MG, Luong TT, Kuechenmeister L, Dunman PM, O'Donnell S, et al. Rbf promotes biofilm formation by Staphylococcus aureus via repression of icaR, a negative regulator of icaADBC. Journal of Bacteriology. 2009;191(20):6363-73.

8. Servant P, Le Coq D, Aymerich S. CcpN (YqzB), a novel regulator for CcpA‐independent catabolite repression of Bacillus subtilis gluconeogenic genes. Molecular Microbiology. 2005;55(5):1435-51.

9. Brinsmade SR, Sonenshein AL. Dissecting complex metabolic integration provides direct genetic evidence for CodY activation by guanine nucleotides. Journal of Bacteriology. 2011;193(20):5637-48.

10. Majerczyk CD, Dunman PM, Luong TT, Lee CY, Sadykov MR, Somerville GA, et al. Direct targets of CodY in Staphylococcus aureus. Journal of Bacteriology. 2010;192(11):2861-77.

11. Kang D-M, Michon C, Morinaga T, Tanaka K, Takenaka S, Ishikawa S, et al. Bacillus subtilis IolQ (DegA) is a transcriptional repressor of iolX encoding NAD+-dependent scyllo-inositol dehydrogenase. BMC Microbiology. 2017;17:1-12.

12. Cho J, Manna AC, Snelling HS, Cheung AL. GraS signaling in Staphylococcus aureus is regulated by a single D35 residue in the extracellular loop. Microbiology Spectrum. 2023;11(5):e01982-23.

13. Liu Q, Yeo W-S, Bae T. The SaeRS two-component system of Staphylococcus aureus. Genes. 2016;7(10):81.

14. Harraghy N, Homerova D, Herrmann M, Kormanec J. Mapping the transcription start points of the Staphylococcus aureus eap, emp, and vwb promoters reveals a conserved octanucleotide sequence that is essential for expression of these genes. Journal of Bacteriology. 2008;190(1):447-51.

15. Ji Q, Chen PJ, Qin G, Deng X, Hao Z, Wawrzak Z, et al. Structure and mechanism of the essential two-component signal-transduction system WalKR in Staphylococcus aureus. Nature Communications. 2016;7(1):11000.

16. Hainzl T, Bonde M, Almqvist F, Johansson J, Sauer-Eriksson A E. Structural insights into CodY activation and DNA recognition. Nucleic Acids Research. 2023;51(14):7631-48.

17. Cotton FA, Hazen Jr EE, Legg MJ. Staphylococcal nuclease: Proposed mechanism of action based on structure of enzyme—thymidine 3′, 5′-bisphosphate—calcium ion complex at 1.5-Å resolution. Proceedings of the National Academy of Sciences. 1979;76(6):2551-5.
